# Interleukin-2-Mediated Engraftment of Human Peripheral Blood Mononuclear Cells in Immunodeficient Mice to Develop a Model of HIV Infection: New Criteria for Engraftment Monitoring

**DOI:** 10.3390/ijms27146266

**Published:** 2026-07-14

**Authors:** Aleksey M. Nagornykh, Marina A. Tyumentseva, Aleksandr I. Tyumentsev, Leonid A. Fedotov, Konstantin S. Karbyshev, Lubov S. Danilova, Andrey S. Akinin, Vasiliy G. Akimkin

**Affiliations:** Central Research Institute for Epidemiology of the Federal Service for Supervision of Consumer Rights Protection and Human Welfare, Moscow 111123, Russiafedotov@cmd.su (L.A.F.); karbyshev@cmd.su (K.S.K.); danilova.l@cmd.su (L.S.D.); akinin@cmd.su (A.S.A.);

**Keywords:** graft-versus-host disease, peripheral blood mononuclear cells, T-helper cells, immune-inflammation indices, human immunodeficiency virus

## Abstract

Graft failure and graft-versus-host disease are the main limiting factors for the engraftment of not only xenogeneic but also allogeneic grafts. Long-term engraftment of human immune cells in mice with severe combined immunodeficiency and *IL2rg* deletion is key to the success of long-term studies in modeling human immunodeficiency virus (HIV) infection. Human peripheral blood mononuclear cells were injected intravenously into males of one strain and females of two other strains of immunodeficient mice. Before the injection of human cells, mice of each strain were irradiated with three different doses. Interleukin-2 was administered to 50% of mice within 5 days of human cell injection. Its effect was assessed by monitoring graft-versus-host disease, survival, complete blood count (CBC) indices, quantification of human cells in whole blood, pathological changes, and immunohistochemical detection of human cells in tissues. Furthermore, the applicability of correlating immune-inflammation indices with mouse body weight (BW) dynamics, graft-versus-host disease (GVHD) progression, degrees of chimerism and human T-helper cells was determined for the first time. As a result of this work, a panel of biomarkers was developed for predicting the long-term welfare of mice with elements of the human immune system (HIS) in long-term studies, including HIV testing.

## 1. Introduction

For relevant modeling of chronic diseases, including infectious diseases such as HIV infection or viral hepatitis, the duration of observation is a primary characteristic. The development of the HIS-mice is a prerequisite for the development and testing of antiretroviral therapy agents and protocols. However, even in humans, engraftment of allogeneic grafts still requires careful immunocorrection and control of GVHD. Conversely, failure or hypofunction of the graft itself can lead to inadequate humanization, although lethal GVHD does not develop. In humans, one of the primary graft failure scenarios is the regeneration of the recipient’s absolute neutrophil count in the absence of donor chimerism. Secondary graft failure is characterized by recipient cytopenia with loss of donor hematopoiesis, developing after introduction of a graft with high donor chimerism [1]. Thus, both the welfare of the animals themselves and the viability of the grafts are of key importance.

The most commonly used mice for the generation of HIS-mice are NSG, NOG, or NRG mice [2,3,4]. Due to the knockout of the *Il2rg* gene, they lack some of the cytokine transduction pathways that lead to a deficiency of functional natural killer cells. This beneficial property allows for the delay of the development of severe GVHD. At the same time, the administration of small doses of interleukin-2 (IL-2_ad_) specifically restores the homeostasis of regulatory T cells in both mice and humans, including those with chronic GVHD [5,6]. Furthermore, small doses of IL-2 allow for an increase in the survival time of allogeneic grafts in HIS-mice [2]. However, due to the short half-life of exogenous IL-2, the effect is short-lived, even with daily injections for several days in a row [7]. On the other hand, immunodeficient mice express their own major histocompatibility complex (MHC), which, upon detection of human leukocyte antigen (HLA), triggers MHC-HLA-mediated immune mechanisms [3,4]. In addition, low doses of IL-2 stimulate the peripheral expansion of recipient CD4^+^ and CD8^+^ regulatory T-cells, promoting the progression of GVHD, since human IL-2 similarly stimulates the proliferation of murine T-cells [2,8]. Therefore, it was important for us to determine the impact of IL-2 administration on both engraftment and GVHD progression.

The HIS-mice, which were engrafted with human peripheral blood mononuclear cells (hPBMCs-mice), achieved rapid reconstitution of human immune cells. However, xenogeneic GVHD develops relatively early in mice [9]. Despite this, hPBMCs-mice are simpler to use, and sufficient concentrations of circulating human cells were achieved 14–21 days post-injection (d.p.i.). These checkpoints are commonly used for experimental HIV infection. In addition, 21 days is the minimum observation window for determining the dynamics of circulating hCD4^+^ T cell concentrations in the blood of mice after HIV infection. Thus, 42 d.p.i. is a sufficient time period for observation when modeling HIV infection in hPBMCs-mice.

HIV primarily infects hCD4^+^ T cells, so the hCD4^+^: hCD8^+^ T cells ratio is one of the factors used to assess the readiness of human cells for in vivo infection [10]. Manipulations with HIV-infected animals require strict biosafety regulations and highly qualified personnel. Therefore, it is important to ensure that the welfare of the HIS-mice allows for the completion of the study within the established timeframe, the grafts are able to maintain their integrity, and GVHD does not critically impact the results.

In most xenograft studies, GVHD in mice is assessed using indirect signs such as BW loss, posture changes, decreased mobility, deterioration in fur quality, etc. [11]. These are the most accessible monitoring methods and do not require sophisticated equipment. However, they provide only a limited understanding of the severity of GVHD. Therefore, clinical signs of GVHD may be identified late or misinterpreted [12,13]. Laboratory test results allow for a more accurate assessment of the welfare of animals. Therefore, identifying signs of graft hypofunction and GVHD progression using CBC indices in mice is also of paramount importance for long-term xenograft studies.

Monocytosis can be a reliable criterion for determining the likelihood of developing chronic GVHD, and thrombocytopenia is one of the common complications of chronic GVHD in humans [14,15]. The role of granulocytes, in particular neutrophils, in GVHD is highly controversial. Although the severity of GVHD in the human intestine has a strong correlation with the number of neutrophils in GVHD foci, neutrophils do not induce GVHD directly but cause tissue damage that promotes T-cell activation, which leads to the progression of GVHD [16]. However, CBC indices are not the only criteria for determining pathology. In humans, engraftment of allogeneic hematopoietic stem cells (HSCs) can be determined by the restoration of the absolute neutrophil count and chimerism analysis, independently of other CBC indices [17,18].

In recent years, the monocyte-to-lymphocyte ratio (MLR), lymphocyte-to-monocyte ratio (LMR), granulocyte-to-lymphocyte ratio (GLR), and platelet-to-lymphocyte ratio (PLR) have been increasingly proposed as diagnostic markers of surgical stress in oncosurgery, in the diagnosis and prognosis of the course of oncological and other diseases, including infectious diseases and GVHD, as well as before allogeneic HSC engraftment [19,20,21,22,23,24,25,26,27,28,29,30,31,32]. Given the existing results of meta-analyses, these immune-inflammation indices may be significant for human and veterinary medicine. However, they have not previously been used to assess the welfare of animal models.

Therefore, we determined the correlation probability between CBC indices, MLR, LMR, GLR, PLR, degree of chimerism (Ch), hCD3^+^hCD4^+^ cell counts (hT-helper cells), BW dynamics, and GVHD progression. Another objective was to determine the feasibility of humanization of NOD-Prkdc*^scid^*Il2rg*^em1^*/Smoc (NSG) mice using hPBMCs and to evaluate the chimerization potential of NOD/ShiLtJGpt-Prkdc*^em26Cd52^*Il2rg*^em26Cd22^*/Gpt (NCG) mice relative to NOD/Shi-Prkdc*^scid^*Il2rg*^em1^*/Cyagen (C-NKG) mice. Thus, we identify critical points and select optimal conditions for reconstitution of the human immune system in the appropriate mouse strain. The most effective protocol will be used to develop HIS-mice, which will be infected with HIV for further research.

## 2. Results

### 2.1. Effects of the Irradiation Dose and IL-2_ad_ on GVHD Progression

**Figure 1:**
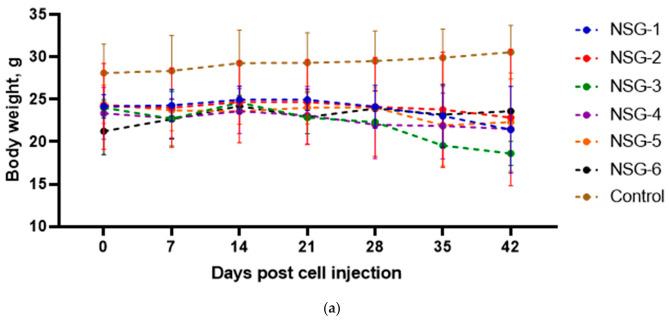

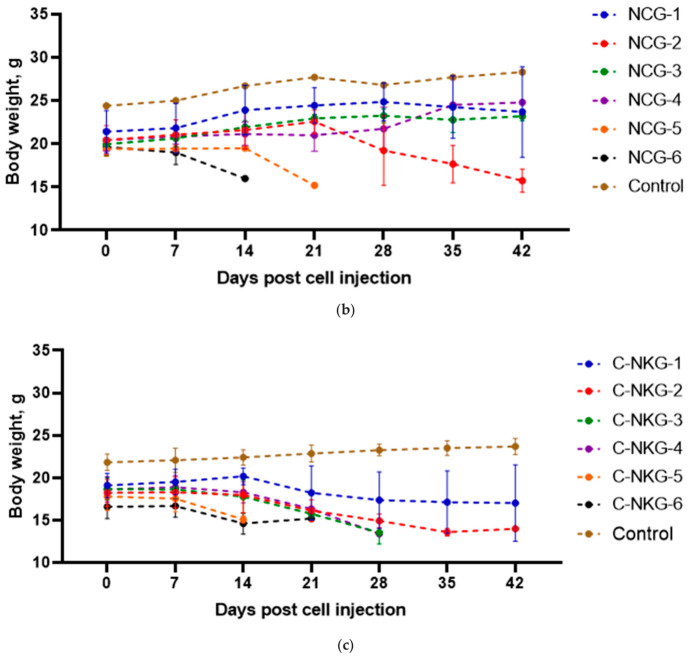
Effect of IrD and IL-2_ad_ on the dynamics of BW: (**a**) in NSG mice; (**b**) in NCG mice; (**c**) in C-NKG mice.

BW dynamics in NSG mice were stable throughout the observation period but were lower than in control animals (Figure 1a). In females, the most pronounced BW loss was observed in NCG-2 and C-NKG-2 mice, despite minimal irradiation dose (IrD).

The two-way analysis of variance (ANOVA) did not confirm the effect of humanization conditions or IL-2_ad_ on BW dynamics for any of the mouse strains. However, Tukey’s multiple comparison test (Tukey’s test) determined the effect of IL-2_ad_ and IrD in NCG-1 and NCG-2 mice, as well as NCG-2 and NCG-4 mice, starting at 28 d.p.i.

**Figure 2:**
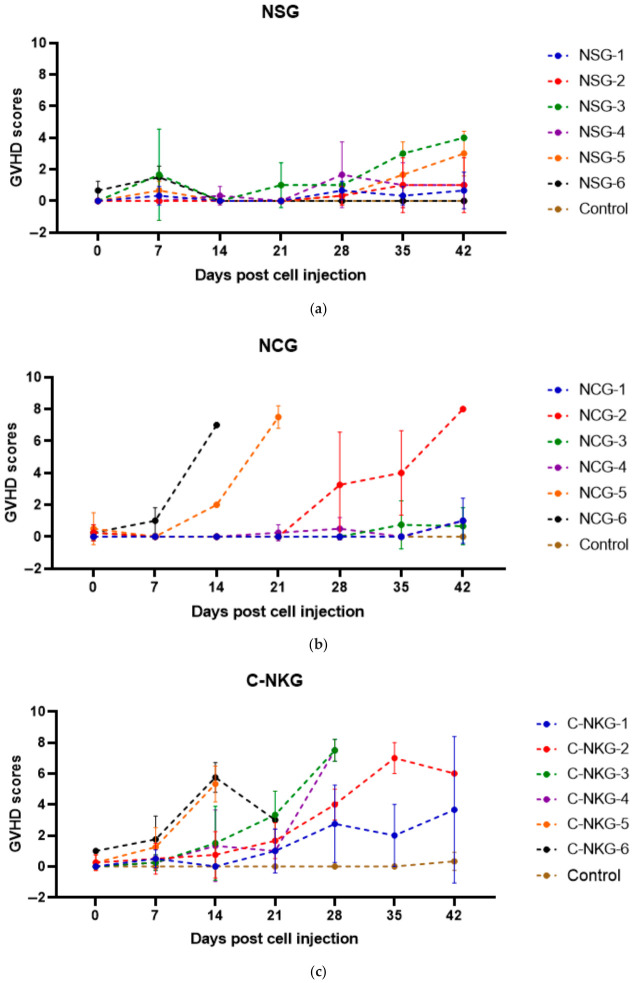
Effect of the IrD and IL-2_ad_ on the dynamics of GVHD: (**a**) in NSG mice; (**b**) in NCG mice; (**c**) in C-NKG mice.

The progression of GVHD in NSG mice was mild (Figure 2a), whereas rapid progression of GVHD was observed in NCG-2 and C-NKG-2 mice at 21 d.p.i. (Figure 2b,c).

ANOVA demonstrated a statistically significant relationship between GVHD progression and humanization conditions in females, but not in males (Table 1). Tukey’s test confirmed the effect of IrD on GVHD progression in NCG mice at 28 d.p.i. and in NSG mice at 35 d.p.i. The effect of IL-2_ad_ was determined only in NCG mice at 28 d.p.i.

### 2.2. The Survival of HIS-Mice Depends on the IrD and IL-2_ad_

**Figure 3:**
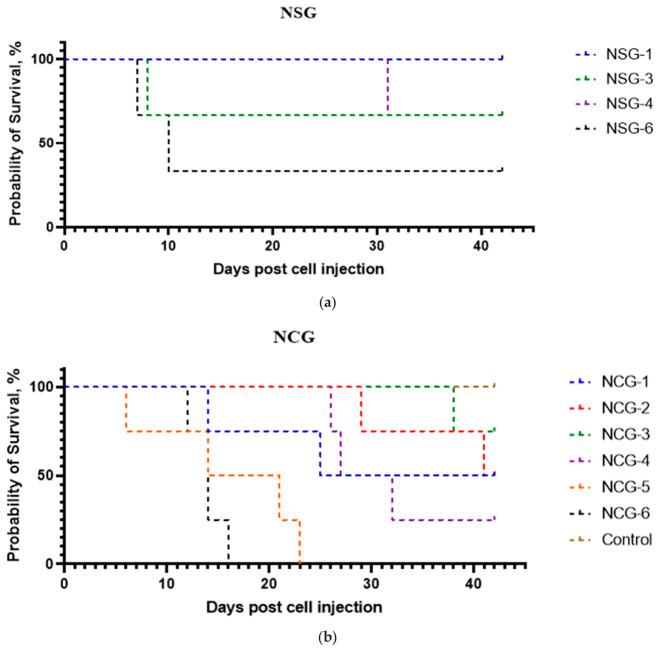

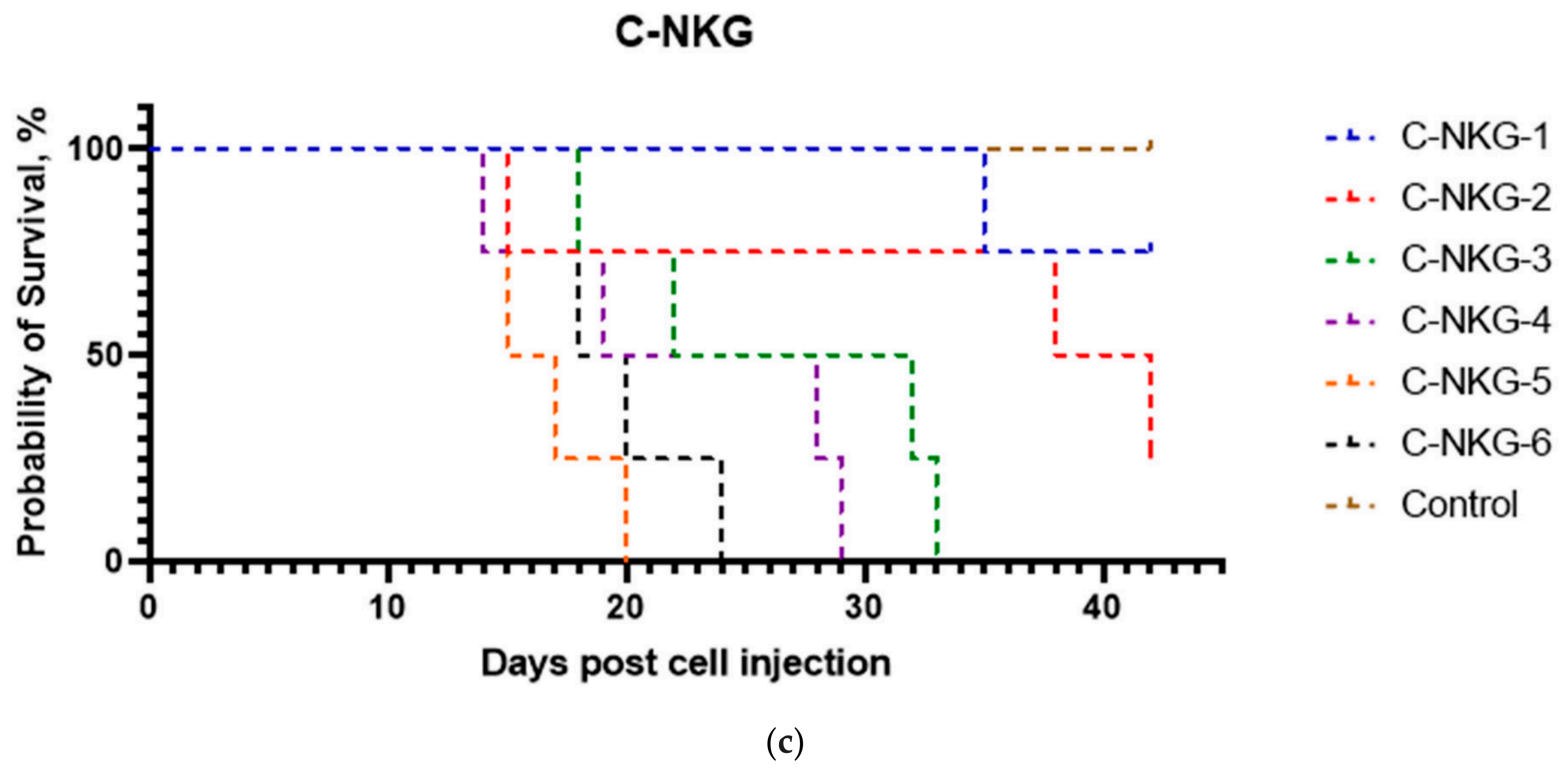
Effect of the IrD and IL-2_ad_ on survival probability: (**a**) in NSG mice; (**b**) in NCG mice; (**c**) in C-NKG mice.

During the observation period, one NSG-3 mouse and one NSG-4 mouse, and two NSG-6 mice were euthanized due to the development of the lethal GVHD phenotype (Figure 3a). However, the mean survival of females was lower. A total of 100% of NCG-5, NCG-6, C-NKG-3, C-NKG-4, C-NKG-5, and C-NKG-6 mice (Figure 3b,c); 75% of NCG-4 and C-NKG-2 mice, 50% of NCG-1 and NCG-2 mice; and 25% of NCG-3 and C-NKG-1 mice were euthanized prematurely.

Survival analysis using the nonparametric Kaplan–Meier method, modified by the Mantel–Cox and Gehan–Breslow–Wilcoxon tests, as well as the log-rank test for trend, did not confirm the effect of IrD and IL-2_ad_ on the probability of survival in NSG mice but did confirm it in NCG mice. In C-NKG mice, the presence of an association was not confirmed by the log-rank test (Table 2).

The mean survival of NSG-1, NSG-2, NSG-3, NSG-4, NSG-5, NCG-3, and C-NKG-1 mice was not determined.

### 2.3. IrD and IL-2_ad_ Determine the Dynamics of hPBMCs Engraftment in C-NKG Mice but Not the Dynamics of hT-Helper Cell Concentration

**Figure 4:**
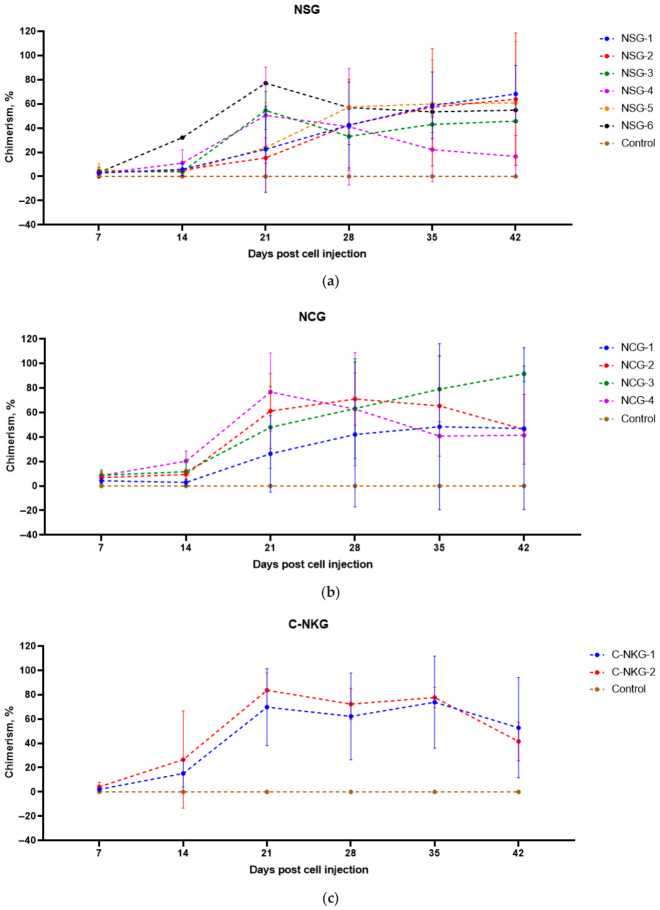
Effect of IrD and IL-2_ad_ on Ch: (**a**) in NSG mice; (**b**) in NCG mice; (**c**) in C-NKG mice.

In the vast majority of mice, an explosive growth of human cell populations was observed from 7 to 21 d.p.i. (Figure 4). The highest Ch was observed in NCG-3 mice. However, Ch was most stable in NSG mice, while a decrease was observed in C-NKG mice at the end of the study.

Despite this, ANOVA revealed no relationship between IrD, IL-2_ad_, and Ch in NSG and NCG mice but confirmed this in C-NKG mice. Tukey’s test revealed no interaction between IrD, IL-2_ad_, and Ch in NSG mice. For the other strains, this relationship was only observed relative to controls.

**Figure 5:**
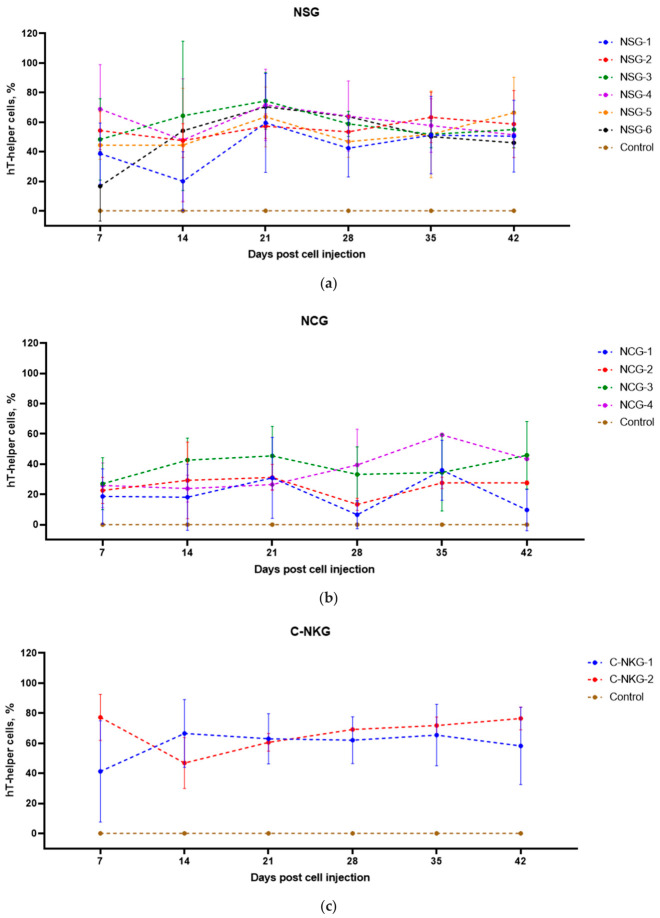
Effect of the IrD and IL-2_ad_ on dynamics of Th: (**a**) in NSG mice; (**b**) in NCG mice; (**c**) in C-NKG mice.

Relatively stable mean hT-helper cells (Th) values were observed in the last 7 days of the study in NSG and C-NKG mice. In NSG mice, it was ~45–65%; in C-NKG mice, ~55–75%; and in NCG mice, ~10–45%. A positive effect of IL-2_ad_ on increasing the number of hCD3^+^hCD4^+^ cells in NSG and C-NKG mice was observed throughout almost the entire study (Figure 5a,c).

The mean maximal Ch value was observed in C-NKG-2 mice and amounted to ~77%. Similar values were achieved in NSG-3 and NSG-4 mice at 21 d.p.i. ANOVA did not confirm the relationship between IrD, IL-2_ad_, and dynamics of Th. Tukey’s test determined the effect of IL-2_ad_ on Th in NSG-1 and NSG-2 mice only at 7 d.p.i.

### 2.4. Analysis of the Dynamics of CBC Indices

#### 2.4.1. Leukocyte Indices

Dynamics of absolute white blood cell count (WBC) in HIS-mice were virtually identical to those in the corresponding control mice (Figure S2a–c). Single peaks of leukocytosis were distinct in NSG and NCG mice, but they did not affect the overall picture. Moderate leukocytosis was observed in C-NKG-1 mice from 14 to 35 d.p.i., but by the end of the study, WBC was similar to that in other C-NKG mice. ANOVA confirmed a statistically significant relationship between IrD, IL-2_ad_, and dynamics of WBC only in NCG mice (Table 3). Tukey’s test revealed the effect of IL-2_ad_ on WBC in C-NKG mice at 35 d.p.i. and in NCG mice at 42 d.p.i.

Dynamics of absolute lymphocyte count (Lymph#) and absolute monocyte count (Mon#) resembled those of WBC. High peak values were observed in individual mice, primarily at 21 d.p.i. ANOVA demonstrated a statistically significant relationship between IrD, IL-2_ad_, and Lymph# dynamics in NCG mice. Tukey’s test revealed a significant effect of IL-2_ad_ on Lymph# dynamics in C-NCG mice that were irradiated with minimal IrD at 42 d.p.i.

ANOVA demonstrated a statistically significant relationship between IrD, IL-2_ad_, and Mon# dynamics in the blood of NCG mice. Tukey’s test revealed significant results between IL-2_ad_ and Mon# dynamics in NCG and C-NKG mice that were irradiated with minimal IrD at 42 and 35 d.p.i., respectively.

Progressive absolute granulocytosis was observed in all HIS-mice, but it was most pronounced in NSG mice. ANOVA results demonstrated no relationship between IrD, IL-2_ad_, and absolute granulocyte count (Gran#) dynamics. However, for NCG and C-NKG mice, which were irradiated with minimal IrD, a relationship between IrD, IL-2_ad_, and dynamics of Gran# was detected at 42 and 28 d.p.i., respectively, using Tukey’s test.

Dynamics of relative lymphocyte count (Lymph%) in NSG mice were characterized by a random alternation of prolonged peaks and troughs, while in NCG and C-NKG mice they were more structured and regular. In both cases, lymphocytosis peaked at 14 d.p.i. Then, in most mice, there was a gradual decline until the end of the study. ANOVA demonstrated a statistically significant relationship between IrD, IL-2_ad_, and dynamics of Lymph% only in C-NKG mice. Other identified interaction terms were also established using Tukey’s test relative to control mice, except for NCG-2 and NCG-4 mice, which allowed us to determine the effect of IrD on dynamics of Lymph% at 21 d.p.i.

The dynamics of the relative monocyte count (Mon%) were linear and varied slightly both within and between strains, ranging from 0 to 5% throughout the study. ANOVA demonstrated a statistically significant relationship between IrD, IL-2_ad_, and dynamics of Mon% in C-NCG mice. Tukey’s test determined the relationship relative to control mice at 14 d.p.i.

Relative granulocyte count (Gran%) peaked between 21 and 28 d.p.i. in most mice. Only C-NKG mice showed moderate relative granulocytosis throughout the study. ANOVA demonstrated a statistically significant relationship between IrD, IL-2_ad_, and dynamics of Gran% in NSG mice. In NCG mice, Tukey’s test revealed a relationship between IrD and dynamics of Gran% at 7 d.p.i.

#### 2.4.2. Erythrocyte Indices

Despite some variability between groups, dynamics of absolute red blood cell count (RBC), hemoglobin count (HGB), and hematocrit (HCT) showed a general trend toward gradual decline throughout the study (Figure S3a–i). ANOVA demonstrated no statistically significant relationship between IrD; IL-2_ad_; and dynamics of RBC, HGB, and HCT. Tukey’s test revealed a relationship only for C-NKG HIS-mice compared to controls.

The mean corpuscular volume (MCV) in all NSG HIS-mice was higher than in controls, while in NCG HIS-mice, this was observed only in those irradiated with 1.5 Gy. In C-NKG HIS-mice, MCV decreased relative to controls from 21 to 28 d.p.i. ANOVA demonstrated a statistically significant relationship between IrD; IL-2_ad_; and dynamics of MCV, mean corpuscular hemoglobin (MCH), and mean corpuscular hemoglobin concentration (MCHC) in NSG and NCG mice. Tukey’s test revealed a significant relationship between IrD and dynamics of MCV and MCH at 28 d.p.i. in NCG mice; in NSG mice that were irradiated with the maximal IrD, a relationship between IL-2_ad_ and dynamics of MCH was determined at 21 d.p.i. In NCG mice, there was a relationship between IrD and dynamics of MCHC at 28 and 35 d.p.i., and between IL-2_ad_ and dynamics of MCHC at 28 d.p.i.; in NSG mice, there was a relationship between IrD and dynamics of MCHC at 28 and 42 d.p.i.

As a rule, red cell distribution width (RDW) was higher in HIS-mice than in controls. ANOVA demonstrated a statistically significant relationship between IrD, IL-2_ad_, and dynamics of RDW in NSG and NCG mice. Tukey’s test for NCG mice revealed a relationship between IrD and dynamics of RDW at 21, 28, and 42 d.p.i., and between IL-2_ad_ and dynamics of RDW at 42 d.p.i.

#### 2.4.3. Platelet Indices

Absolute platelet count (PLT) in HIS-mice was lower than in controls throughout the study (Figure S4a–c). ANOVA did not confirm a statistically significant relationship between IrD, IL-2_ad_, and dynamics of PLT, while Tukey’s test revealed a relationship between NSG and C-NKG in HIS-mice compared to controls.

The dynamics of mean platelet volume (MPV) demonstrated a trend opposite to that of platelets, indicating a significant impact of humanization on thrombopoiesis. Throughout virtually the entire study, all HIS-mice demonstrated higher values than controls. ANOVA did not confirm a statistically significant relationship between IrD, IL-2_ad_, and dynamics of MPV. Tukey’s test determined a relationship between IrD and dynamics of MPV in NCG mice at 28 d.p.i. and between IL-2_ad_ and dynamics of MPV in C-NKG mice at 42 d.p.i.

The platelet distribution width (PDW) was within virtually identical ranges in all mice. ANOVA demonstrated no statistically significant relationship between IrD, IL-2_ad_, and dynamics of PDW. Tukey’s test determined the relationship between IL-2_ad_ and dynamics of PDW at 35 d.p.i.

Since PLT directly determines plateletcrit (PCT), its dynamics in HIS-mice were lower than in controls. Therefore, ANOVA demonstrated the absence of a statistically significant relationship between IrD, IL-2_ad_, and dynamics of PCT. Tukey’s test determined the relationship between IL-2_ad_ and dynamics of PCT in NSG mice at 35 d.p.i. and between IrD and dynamics of PCT in NCG mice at 42 d.p.i. (Table 3).

### 2.5. Immune-Inflammation Indices

**Figure 6:**
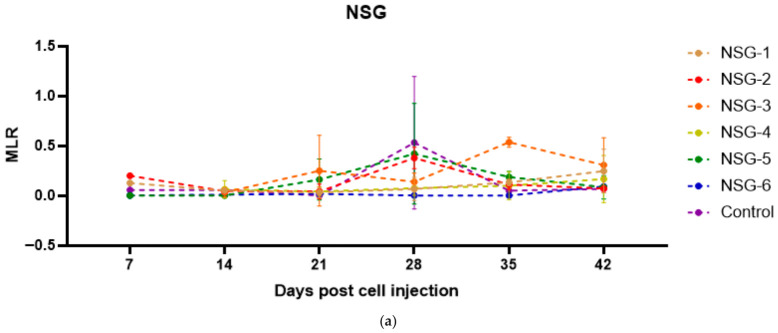

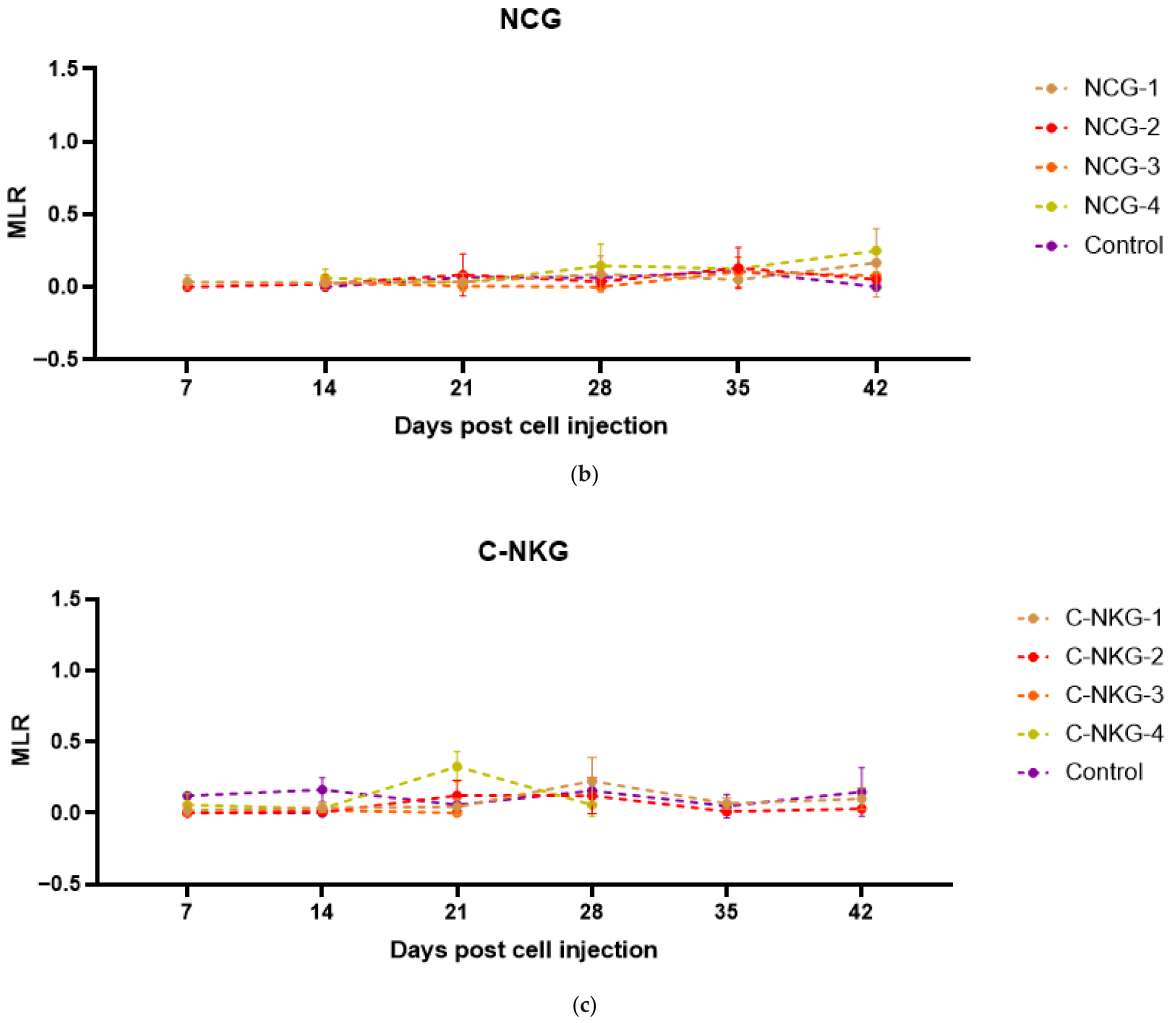
Effect of the IrD and IL-2_ad_ on dynamics of MLR: (**a**) in NSG mice; (**b**) in NCG mice; (**c**) in C-NKG mice.

Dynamics of MLR in NSG HIS-mice were mostly undulating (Figure 6a), whereas in females, they were more linear and within the control values (Figure 6b,c).

**Figure 7:**
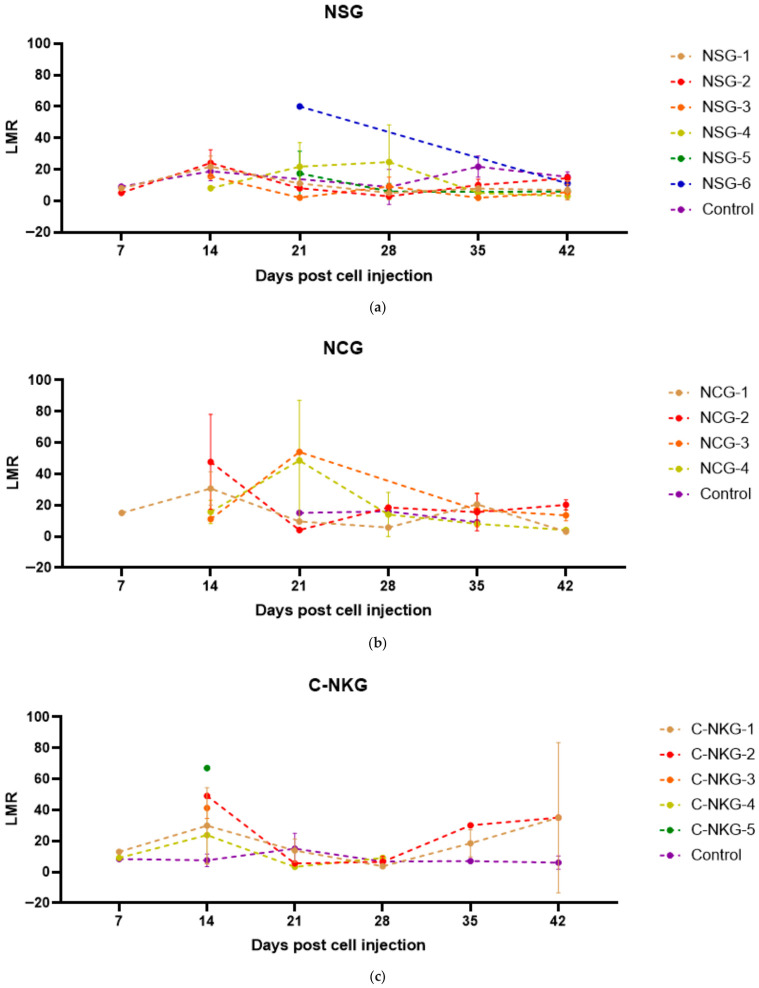
Effect of the IrD and IL-2_ad_ on dynamics of LMR: (**a**) in NSG mice; (**b**) in NCG mice; (**c**) in C-NKG mice.

The dynamics of LMR were more variable, but due to the absence of monocytes in many mice during the first weeks after myeloablation, some values were not determined (Figure 7).

**Figure 8:**
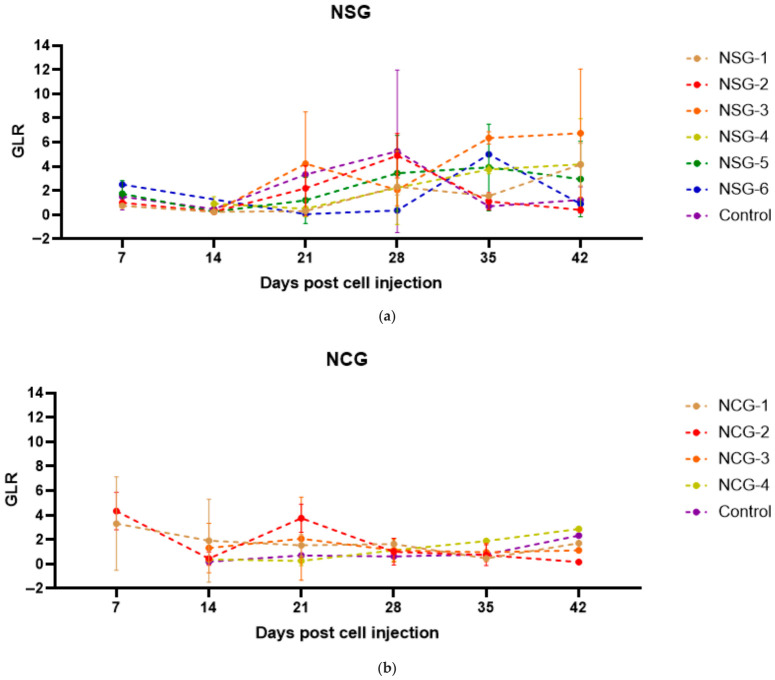

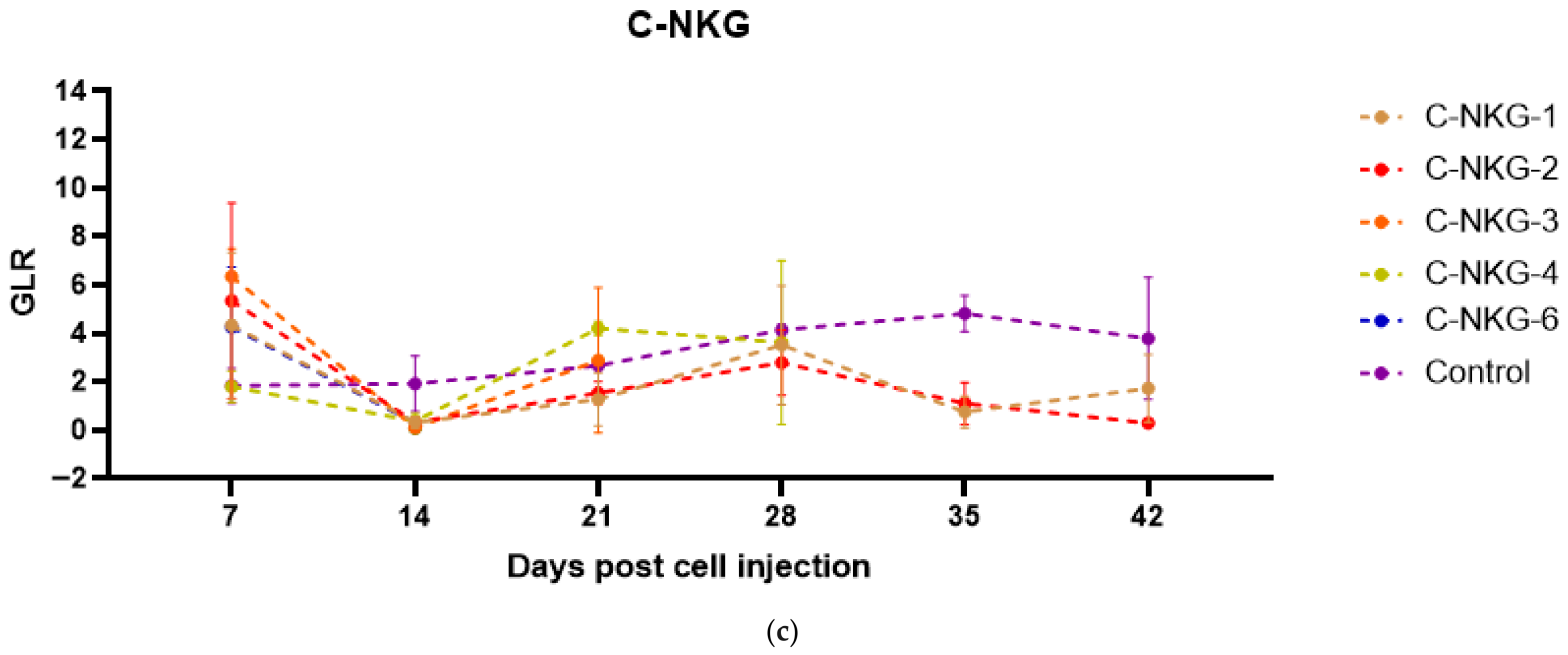
Effect of the IrD and IL-2_ad_ on dynamics of GLR: (**a**) in NSG mice; (**b**) in NCG mice; (**c**) in C-NKG mice.

The dynamics of GLR were also highly variable. In NSG and C-NKG mice, they were undulating, while in NCG mice, they were more linear (Figure 8).

**Figure 9:**
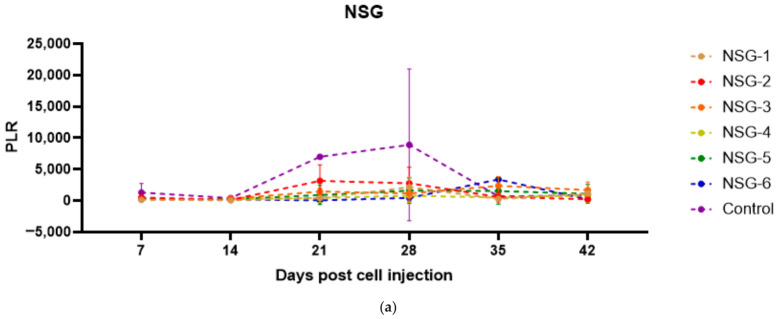

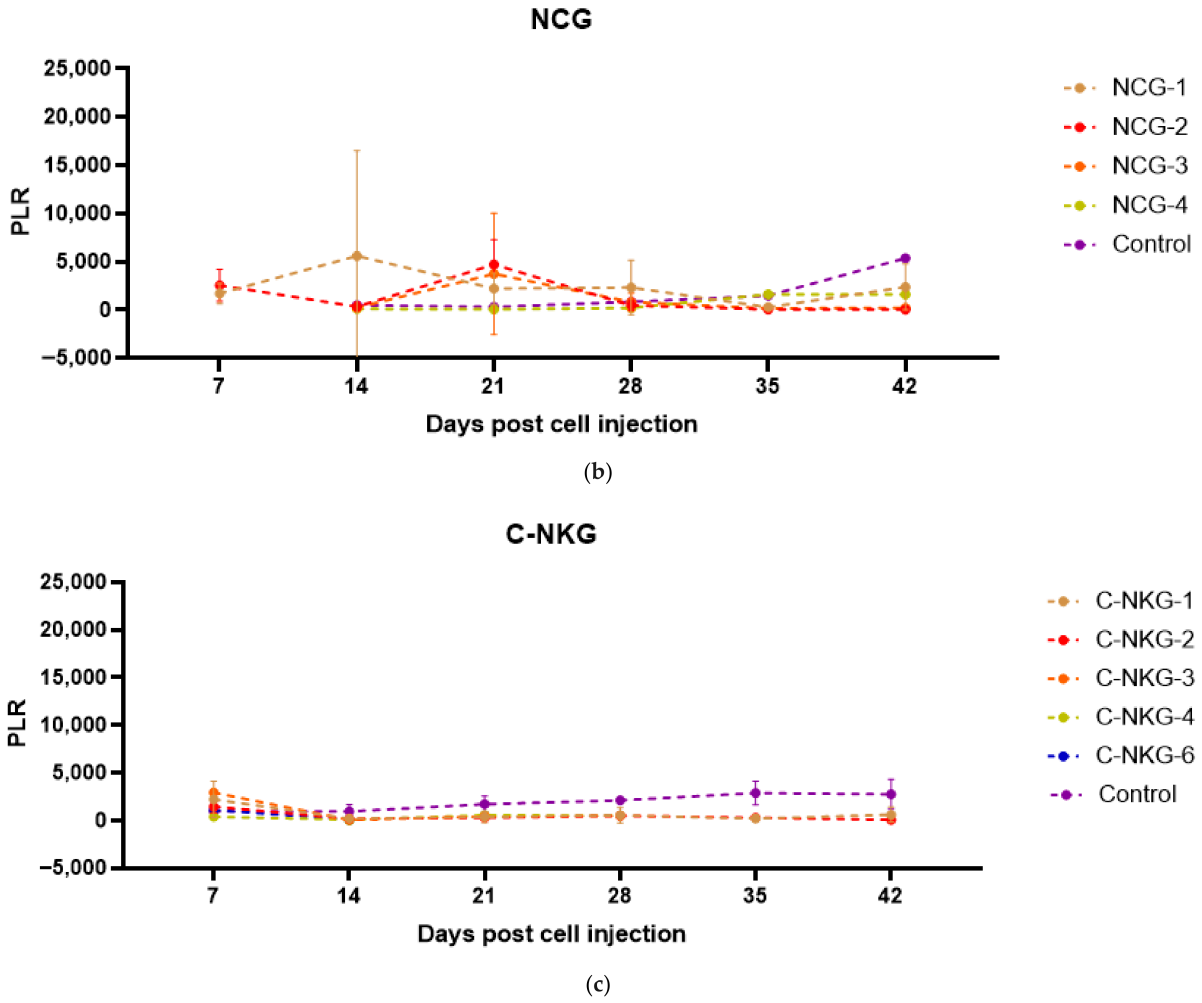
Effect of the IrD and IL-2_ad_ on dynamics of PLR: (**a**) in NSG mice; (**b**) in NCG mice; (**c**) in C-NKG mice.

The dynamics of PLR in HIS-mice were generally linear and differed little from the controls (Figure 9).

Thus, a comparative analysis of the dynamics of immune-inflammation indices did not yield significant results. However, determining the degree of their correlation with key characteristics of mouse welfare may have prognostic value.

### 2.6. The Relationship of the Main Characteristics of HIS-Mice Welfare with Each Other and Immune-Inflammation Indices

Determining a reference range requires a large sample size. Therefore, both the indices themselves and their correlations with key welfare indicators of humanized mice were calculated using Spearman’s coefficient. Determining a linear correlation can indicate a direct relationship between two parameters. Given the growing popularity of personalized medicine, the correlation was determined individually for each mouse. To better understand the reasons for the shortened observation window, a correlation was determined for each mouse strain for both those euthanized upon reaching humane endpoints (EUTH) and those surviving to the end of the study (SURV).

The starting control value for HIV introduction into HIS-mice can be the concentration of hCD45^+^ cells in peripheral blood, or the hCD4^+^: hCD8^+^ T cell ratio, or the expiration of a certain period of time from the moment of human cell introduction (usually 14–21 d.p.i.) [10,33,34]. Therefore, the identification of prognostic factors for the duration of observation of infected mice will help in planning a long-term observation.

The main characteristic influencing HIS-mice survival is GVHD progression, which is based on scores for BW reduction, fur and skin quality, and changes in mobility and posture. In the absence of GVHD, correlation calculations are not feasible due to the impossibility of violating mathematical rules. Therefore, determining a linear relationship between parameters was only possible in mice exhibiting signs of GVHD. Thus, it was determined that BW had a very high negative correlation with GVHD in 92% of NSG_SURV_ mice; 80% of NCG_SURV_ mice; and 100% of C-NCG_SURV_, NCG_EUTH_, and C-NCG_EUTH_ mice (Figures S5 and S6).

Other important characteristics in modeling HIV infection are Ch and Th. A negative BW/Ch correlation was detected in 71% of NSG_SURV_, 80% of C-NKG_SURV_, and 89% of NCG_EUTH_ and C-NKG_EUTH_ mice, while the BW/Th correlation was positive in 71% of NSG_SURV_ mice and negative in 80% of C-NKG_SURV_ mice.

A positive GVHD/Ch correlation was detected in 75% of NSG_SURV_ mice and 100% of NCG_SURV_, C-NKG_SURV_, NCG_EUTH_, and C-NKG_EUTH_ mice, while 75% of NSG_SURV_ mice and 100% of C-NKG_SURV_ mice had a positive GVHD/Th correlation (Figures S5 and S6). The Ch/Th correlation was negative in 75% of C-NKG_EUTH_ mice (Figure S6b).

The most pronounced negative BW/WBC correlation was observed in 80% of C-NKG_SURV_ mice, while it was positive in 88% of C-NKG_EUTH_ mice. As demonstrated above, BW/GVHD have a very high negative correlation; accordingly, 100% of C-NKG_SURV_ mice had a highly positive GVHD/WBC correlation, while 75% of C-NKG_EUTH_ mice had a very high negative correlation (Figures S7 and S8).

Also, a high negative BW/Lymph# correlation was found in 80% of C-NKG_SURV_ and NCG_EUTH_ mice, while a negative GVHD/Lymph# correlation was detected in 75% of NSG_SURV_ mice and a positive one in 100% of C-NKG_SURV_ mice (Figures S7 and S8). A high negative BW/Mon# correlation characterized 80% of C-NKG_SURV_ mice, while a high positive GVHD/Mon# correlation corresponded to 75% of NCG_SURV_ mice and 100% of C-NKG_SURV_ mice. In addition, a high positive GVHD/Gran# correlation was found in 75% of NCG_SURV_ mice and 75% of C-NKG_SURV_ mice.

A high positive BW/RBC correlation was detected in 100% of C-NKG_SURV_ and 75% of C-NKG_EUTH_ mice, while a high negative GVHD/RBC correlation was found in 75% of C-NKG_SURV_, 86% of NCG_EUTH_, and 88% of C-NKG_EUTH_ mice. High and very high positive BW/PLT correlations were found in 75% of NCG_SURV_, 100% of C-NKG_SURV_, 100% of NCG_EUTH_, and 100% of C-NKG_EUTH_ mice, while a high negative GVHD/PLT correlation was found in 100% of C-NKG_SURV_, 100% of NCG_EUTH_, and 100% of C-NKG_EUTH_ mice (Figures S7 and S8).

A high positive Ch/WBC correlation was detected in 86% of NSG_SURV_, 75% of NCG_SURV_, and 80% of C-NKG_SURV_ mice, while a moderate negative Th/Lymph# correlation was detected in 100% of C-NKG_SURV_ mice (Figure S9). In addition, moderate and low positive Ch/Lymph# correlations were detected in 75% of NCG_SURV_ and 100% of C-NKG_SURV_ mice, respectively. A high positive Ch/Mon# correlation was detected in 86% of NSG_SURV_, 75% of NCG_SURV_, and 80% of C-NKG_SURV_ mice, while a moderate positive Th/Mon# correlation was detected in 80% of C-NKG_SURV_ mice (Figure S9). A high positive Ch/Gran# correlation was found in 79% of NSG_SURV_, 75% of NCG_SURV_, and 100% of C-NKG_SURV_ mice, while a moderate positive Th/Gran# correlation was found in 100% of C-NKG_SURV_ mice.

High and very high negative Ch/RBC correlations were observed in 100% of NCG_EUTH_ mice, while moderate positive Th/RBC correlations were observed in 78% of them. A very high negative Ch/PLT correlation was detected in 89% of NCG_EUTH_ mice and 88% of C-NKG_EUTH_ mice (Figure S10).

A positive Ch/MLR correlation was observed in 86% of NSG_SURV_, 88% of NCG_SURV_, and 100% of C-NKG_SURV_ mice, while a high and very high negative Ch/LMR correlation was characteristic of 75% of NSG_SURV_, 100% of NCG_SURV_, and 75% of C-NKG_SURV_ mice, and a moderate positive Th/LMR correlation was characteristic of 75% of C-NKG_SURV_ mice. High and moderate positive Ch/GLR correlations were observed in 71% of NSG_SURV_ and 80% of C-NKG_SURV_ mice, respectively. Moreover, a moderate negative Ch/GLR correlation was determined in 80% of C-NKG_SURV_ mice, while a moderate positive Ch/PLR correlation was characteristic of 75% of NCG_SURV_ mice (Figure S11).

A high positive BW/LMR correlation was observed in 83% of NSG_SURV_ mice, and a high negative correlation was observed in 75% of C-NKG_SURV_ mice, while 100% of NCG_SURV_, NCG_EUTH_, and C-NKG_SURV_ mice, respectively, exhibited a high and moderate positive GVHD/MLR correlation (Figures S11 and S12). A very high negative GVHD/LMR correlation was detected in 100% of NCG_SURV_ mice.

A high negative BW/GLR correlation was characteristic of 83% of C-NKG_EUTH_ mice, while a high positive GVHD/GLR correlation was observed in 82% of NSG_SURV_ mice, and a moderately low correlation was observed in 75% of C-NKG_SURV_ mice. Finally, a high positive BW/PLR correlation was detected in 80% of C-NKG_SURV_ mice, and a high negative GVHD/PLR correlation was observed in 100% of these same mice (Table 4).

Thus, this study successfully validated a new panel of biomarkers for predicting the long-term welfare of hPBMCs in long-term studies of HIV infection. Spearman coefficients were determined for all correlation types; however, Table 4 only displays results that generalize to at least 75% of mice of a single strain. The most reliable indicators of long-term welfare of hPBMCs include positive correlations of GVHD/Th, GVHD/Mon#, GVHD/Gran#, Ch/WBC, Ch/Lymph#, Ch/Mon#, and Ch/Gran#.

### 2.7. Macromorphological Analysis

The lowest number of pathologies was detected in NSG mice and was independent of IL-2_ad_. Notably, hyperplasia of the spleen was detected in all NSG-2 mice. Furthermore, the relative brain weight of IL-2_ad_-treated HIS-mice was higher than that of those that were not. ANOVA confirmed a statistically significant relationship between the relative weight of target organs, humanization conditions, and IL-2_ad_ for HIS-mice. Tukey’s test determined the relationship for the relative weight of the liver and small intestine in NSG mice, for liver weight in NCG mice, and for small intestine weight in C-NKG mice.

The most common types of pathologies recorded during macroscopic examination were anemia and atrophy of the organs (Table 5).

Thus, IL-2_ad_ may contribute to the development of severe multiorgan pathology in HIS-mice.

### 2.8. Micromorphological Examination

Microscopic examination of histological bone marrow (BM) specimens from NSG mice irradiated with maximal IrD revealed pronounced atrophy and anemia. Mice treated with IL-2_ad_ had a higher proportion of granulocytic and erythroid cells, confirming its effect on enhancing hematopoiesis. Severe BM atrophy was observed in NSG and NCG mice with minimal IrD and IL-2_ad_. A pronounced increase in hCD4^+^ cell counts was predominant in the BM of mice treated with IL-2_ad_ (Table S1).

In the spleen, a negative effect of moderate and maximal IrD and IL-2_ad_ was also observed. However, in this case, the greater negative effect was associated with parenchymal anemia: in all untreated IL-2_ad_ mice, the red pulp was abundantly filled with erythrocytes. More pronounced atrophy of the white pulp (sometimes with areas of amyloidosis) was characteristic of mice treated with IL-2_ad_. As a rule, hCD4^+^ cells were concentrated at the periphery of the white pulp (Table S2).

Glomerular atrophy was characteristic for mice irradiated with moderate and maximal IrD, and IL-2_ad_ exacerbated the severity of the process. Furthermore, high diffuse infiltration of the perivascular interstitium with hCD4^+^ cells was characteristic of mice treated with IL-2_ad_. The human cells were localized within the glomeruli and also diffusely distributed in the interstitium of the cortex and medulla, as well as in the adipose tissue adjacent to the capsule (Table S3).

Infiltrates of hCD4^+^ cells were also detected in the perivascular interstitium, near the triads, and within the sinusoidal lumens of the liver parenchyma. Pronounced cytosis was noted in NSG and NCG mice that were irradiated with minimal and moderate IrD. These mice also exhibited minor areas of amyloidosis and moderate hemosiderosis of the perivascular interstitium (Table S4).

Examination of the lungs revealed more pronounced cytosis in HIS-mice compared to controls. Extensive hCD4^+^ cell infiltrates were characteristic of mice untreated with IL-2_ad_ NSG. The cells were distributed diffusely throughout the interstitium, and their clusters were more frequently observed in the perivascular and peribronchial interstitium (Table S5).

The morphological patterns of the small and large intestines of HIS-mice and control mice showed no obvious differences (Tables S6 and S7). In HIS-mice, isolated hCD4^+^ cells were scattered throughout the lamina propria and adjacent adipose tissue. However, hCD4^+^ cell counts were more pronounced in mice treated with IL-2_ad_. A similar pattern was observed in the large intestine.

The brain was characterized by a predominance of scattered hCD4^+^ cell infiltrates in HIS-mice untreated with IL-2_ad_ (Table S8).

The morphology of the male reproductive system is more complex than that of females, so comparing their pathological features is inappropriate. Thus, diffuse hCD4^+^ cell infiltrates were detected in the prostate gland, epididymal interstitium, and bulbourethral glands, while in females, they were found in the endometrium (Table S9).

Thus, in the case of successful hPBMCs engraftment, the atrophy of BM and spleen tissue that develops over time directly influences the progression of GVHD. Furthermore, the severity of most blood abnormalities in HIS-mice depends on IrD and IL-2_ad_. For NSG HIS-mice, ANOVA revealed a statistically significant relationship between IrD, IL-2_ad_, and:

- Dynamics of the Gran%, MCV, MCH, MCHC, RDW;
- Target organ mass.

For NCG HIS-mice, ANOVA revealed a statistically significant relationship between IrD, IL-2_ad_, and:

- GVHD progression;
- Survival probability;
- Dynamics of the WBC, Lymph#, Mon#, MCV, MCV, MCH, MCHC, and RDW;
- Target organ weights.

Finally, for C-NKG HIS-mice, ANOVA revealed a statistically significant relationship between IrD, IL-2_ad_, and:

- GVHD progression;
- Survival probability;
- Ch;
- Dynamics of the Lymph% and Mon%;
- Target organ weights.

## 3. Discussion

Although subcutaneous administration of IL-2 has a longer duration of effect, it is undesirable due to the occurrence of a local irritant reaction. In addition, intraperitoneal administration prolongs the activity of IL-2 in the blood compared to intravenous [7]. In a study of the duration of survival of pancreatic grafts in NSG mice, a single low dose of IL-2 of 7.5 IU/g (~225 IU/mouse) and a single high dose of 24.9 IU/g (~747 IU/mouse) were used [2]. At the same time, a single immunotherapeutic dose for humans is 158.8 μg/kg [35]. Considering that we used a shorter course of IL-2_ad_ treatment, a single dose for administration was 5 μg/mouse regardless of the strain.

IL-2_ad_ exerted a prolonged beneficial effect on reconstitution of the HIS in irradiated with 1 Gy NSG and NCG mice, including dynamics of Th in NSG mice. However, irradiation of NCG and C-NKG mice with 2 Gy resulted in the development of a lethal GVHD phenotype and premature euthanasia of all C-NKG HIS-mice, except C-NKG-1; all IL-2_ad_ NCG HIS-mice; and IL-2_ad_ NSG-6 mice.

In our experience, mice exposed to excessive irradiation were euthanized within the first 14 d.p.i. [36]. Significant mortality in mice treated with IL-2_ad_ occurred primarily after 14 d.p.i. (Table S10). Considering that IL-2_ad_ can enhance graft survival in HIS-mice, it was administered within the first 5 d.p.i. On the other hand, endogenous human IL-2 stimulates peripheral expansion of murine CD4^+^ and CD8^+^ regulatory T-cells [2,8]. However, WBC in mice remained low for a long time (Figure S2). Thus, in the IrD/graft concentration pair, IrD plays a leading role in GVHD.

Despite the fact that irradiated mice with high IrD and HIS-mice treated with IL-2_ad_ were demonstrated to have elevated Ch, this led to their premature euthanasia. At the end of the study, NCG and C-NKG HIS-mice had ~50%, while NSG HIS-mice had ~40–70% circulating human cells. Furthermore, NSG-1, NSG-2, NSG-3, and NSG-5 mice showed a tendency toward further increases in Ch.

Although NCG-1 mice received minimal IrD and demonstrated a low Ch throughout the study, they demonstrated comparatively poor survival compared to other strains exposed to similar factors. Mortality occurred between 15 and 25 d.p.i. (Figure 3b). However, NCG mice did not exhibit active regenerative granulocytosis until 28 d.p.i. (Figure S2k). Thus, this acute GVHD may be due to individual partial activation of the recipient immune system, as NCG-1 mice exhibited elevated Gran# and Gran% values at this time (Figure S2k,t).

The concentration of circulating Th susceptible to HIV infection in C-NKG-1 mice was ~50% at the end of the study and showed a tendency to decrease further. In NCG HIS-mice, it was lower (~10–40%) and, except in NCG-3 mice, also tended to decrease over time. In turn, in NSG HIS-mice, the proportion of Th was higher (~45–65%). Notably, HIS-mice untreated with IL-2_ad_ showed a promising tendency to have an increase in the proportion of Th at the end of the study.

Although significant GVHD was observed in NCG-2, C-NCG-1, and C-NCG-2 mice at 35–42 d.p.i., the timing of its development is limited to a short-term study of HIV infection. If HIV is injected at 14–21 d.p.i., complete depletion of hT-helper cells is possible within the next 14–21 days. Thus, even if mice reach humane endpoints, they may coincide with the final checkpoint. Significant GVHD was likely caused by the severe leukocytosis observed in these mice at 42 d.p.i. (Figure S2b,c,e,f,h,i,k,l). Therefore, it should be considered that the activated immune system of recipients may contribute to partial depletion of the human cells, which may be mistaken for the results of HIV infection.

Since modern medicine practices the development of personal avatars for therapy, including infectious diseases, the processing of the study results was carried out in both group and individual formats [37]. Analysis of the mean values for the relative mass of the target organs by group, except for the reproductive tract, demonstrated the predominance of this characteristic in HIS-mice over the controls. This is partly due to the progression of GVHD, since prolonged loss of appetite leads to atrophy of the reproductive organs. The influence of IrD and IL-2_ad_ was also confirmed in a microscopy of hematoxylin and eosin (H&E)-stained histological preparations of BM and spleen. The most pronounced atrophy of BM was observed in mice irradiated with 1.5 Gy and 2 Gy and in mice treated with IL-2_ad_. Localized in internal organs, human cells rarely formed extensive foci of infiltration, except for the interstitium of the liver and lungs. This is a positive sign for mice welfare. Moreover, IL-2_ad_ led to active migration of human cells in the tissues of the BM, intestines, and brain.

Individual statistical analysis demonstrated that in 91% of HIS-mice, regardless of strain, IL-2_ad_, and observation duration, BW had a very high negative correlation with GVHD. Given the increased safety precautions required when working with HIV-injected mice, eliminating all GVHD progression assessment criteria other than dynamics of BW preserves the objectivity of the animal welfare assessment. This eliminates the need to spend time determining posture, mobility, etc. Furthermore, the BW value cannot be zero, making the results of the statistical analysis more informative. Finally, simple randomization is typically performed based on BW, which facilitates the traceability of results [38].

In 82% of HIS-mice, a negative BW/Ch correlation was determined, regardless of IrD, IL-2_ad_, and duration of observation (including 89% of HIS-mice_EUTH_). At the same time, a positive GVHD/Ch correlation was characteristic for 94% of HIS-mice (including 100% of HIS-mice_EUTH_). Thus, a decrease in BW can be considered an indicator of a prospective increase in Ch. Also, GVHD/Th, GVHD/Mon#, GVHD/Gran#, Ch/WBC, Ch/Lymph#, Ch/Mon#, and Ch/Gran# positive correlations can be used as indicators of the long-term welfare of HIS-mice and the graft. In addition, a negative Ch/PLT correlation was characteristic of 89% of HIS-mice_EUTH_, 78% of Ch/RBC, 100% of GVHD/PLT, and 81% of GVHD/RBC. In addition, the negative BW/WBC correlation can be used as a prognostic indicator of long-term welfare of C-NKG HIS-mice.

The correlation of Ch and Th with immune-inflammation indices can be useful with restrictions. Thus, 91% of all HIS-mice_SURV_ had a positive Ch/MLR correlation, while 83% of HIS-mice_SURV_ had a negative Ch/LMR correlation. However, using BW/RBC, BW/PLT, GVHD/Ch, and Ch/Th correlations as prognostic indicators of long-term welfare of HIS-mice is inappropriate.

As a rule, monitoring of the effectiveness of HIV infection is carried out by comparing the dynamics of Th and viral load in infected and control HIS-mice [10,39]. However, it is quite difficult to achieve stable Ch and Th values over a long period of time even in uninfected HIS-mice, given their individual characteristics. In addition, part of the viral load can be reduced by the mouse’s own immune system, which is regenerated even in HIS-mice during long-term observation [36]. In this regard, it seems advisable to supplement the panel of biomarkers for assessing HIV infection in HIS-mice with such calculated indicators as Ch/BW, Ch/WBC, Ch/Lymph#, Ch/Mon#, Ch/Gran#, Ch/MLR, and Th/GVHD. Finally, GVHD, like HIV infection, is a chronic disease, so some of the indicators we propose can be taken into account when developing antiretroviral drugs and protocols [40].

## 4. Materials and Methods

### 4.1. Animals

All manipulations were carried out under sterile conditions. A total of 21 specific-pathogen-free male NOD-Prkdc*^scid^*Il2rg*^em1^*/Smoc mice (Laboratory Animal Nursery of the Institute of Bioorganic Chemistry named after M. M. Shemyakin and Yu. A. Ovchinnikov, Moscow Region, Russia), 28 female NOD/ShiLtJGpt-Prkdc*^em26Cd52^*Il2rg*^em26Cd22^*/Gpt mice (Gempharmatech Co. Ltd., Nanjing, China), and 28 female NOD/Shi-Prkdc*^scid^*Il2rg*^em1^*/Cyagen mice (Cyagen Biosciences Inc., Suzhou, China) aged 5–6 weeks were housed in the ISOcage P bioexclusion system (Techniplast, Buguggiate, Italy) at a temperature of +21 to +23 °C and an air humidity of 45–65%. Animals were maintained under a 12 h day/night cycle.

Feeding was carried out with a standard maintenance diet for rodents (4RF21, Mucedola S.R.L., Settimo Milanese, Italy). Drinking water was provided with filtered tap water. Mice had unlimited access to water and feed. Fine-grained (3 mm fraction) birch chips (LLC Filonichev, Novosibirsk, Russia) were used as bedding. Feed, drinking water, and bedding were sterilized in an autoclave before being given to the animals.

Mice of each strain were randomized into 7 groups using RandoMice v1.1.7 software based on BW. RandoMice software was written in C# using .NET Framework 4.7.2 and Microsoft Visual Studio version 16.4 [41].

Whole-body irradiation of mice was performed 24 h prior to hPBMCs administration using a CIX3 X-ray irradiator (Xstrahl LTD, Walsall, UK) at the IrD indicated in Table 6. Mice were previously induced into a state of general anesthesia by inhalation of an anesthetic mixture based on oxygen and Isoflurane (Laboratorios Karizoo, Barcelona, Spain) using a Biosthesia 300 laboratory animal anesthesia system (Vilber Lourmat, Collégien, France).

### 4.2. Cell Culture

hPBMCs were isolated using Lympholyte-H cell separation medium (Cedarlane Laboratories, Burlington, ON, Canada) from leukocyte concentrates (Blood Center of the Federal Medical and Biological Agency of Russia, Moscow, Russia) of three anonymized healthy donors according to the manufacturer’s recommendations. The isolated hPBMCs were resuspended in fetal bovine serum supplemented with 10% dimethyl sulfoxide and frozen in equal aliquots in cryovials for subsequent storage in liquid nitrogen. On the day of engraftment, hPBMC aliquots were thawed, and the cells were washed three times with 1 × DPBS. To determine the density and viability of cells, as well as the initial proportions of hCD3^+^ and hCD4^+^ populations, the suspension sample was stained with solutions of fluorescent antibodies and viability dye (Table 7) in accordance with the manufacturer’s recommendations. Immunophenotyping was performed using a NovoCyte 3000VYB flow cytometer (Agilent Technologies, Santa Clara, CA, USA).

Based on cell density measurements, the required volume for administration to mice was selected and supplemented with 416 IU/mL IL-2 (11017; NPK BIOTECH, Saint Petersburg, Russia). The cell suspension (0.3 mL) was transferred to sterile microcentrifuge tubes and placed on ice for administration to mice.

We previously demonstrated that GVHD progression in hPBMCs-mice depends on the concentration of engrafted human cells and preliminary myeloablation [36]. Therefore, maximal concentrations of hPBMCs were used to develop the acute GVHD.

### 4.3. Administration of the Cells and IL-2_ad_

Suspensions containing 0.8 ± 0.6 × 10^7^ hPBMCs (0.2 mL) were injected into the lateral tail vein. Control mice were injected with 0.2 mL of 1 × DPBS. Injections were performed using syringes with a 30 G needle (1046152, Vogt Medical GmbH, Karlsruhe, Germany). IL-2_ad_ was administered at 5000 IU/mouse/day for 5 consecutive days, starting from the day of hPBMC administration, according to Table 6.

### 4.4. Evaluation of the Impact of Humanization Conditions and IL-2_ad_ on the Progression of GVHD

Mice were monitored daily for GVHD assessment throughout the study. Mice were examined weekly for the maximal study duration until humane endpoints were reached, according to the adapted method of Seng, A. and Markiewicz, M.A. [11]:

- Mice were deprived of feed and water, and their activity was observed for 5 min. Scoring was as follows: 0 = the mouse began moving within 2 min and continued moving, 1 = the mouse took more than 2 min to stand up and move slowly, and 2 = the mouse did not stand up for 5 min and moved only when touched.
- BW loss: 0 = <10%, 1 = 10–20%, and 2 = ≥20%
- Posture: 0 = no change, 1 = hunched posture at rest, and 2 = hunched posture makes movement difficult.
- Fur texture: 0 = no change, 1 = slight to moderate ruffle, 2 = severe ruffle.
- Skin condition: 0 = no change, 1 = flaking of hairless areas, 2 = obvious flaking of skin in the area of hair loss.

The humane endpoints of the study were:

- Assigning a mouse 7 or more scores;
- BW loss by more than 20%.

If humane endpoints were not reached, mice were euthanized at 42 d.p.i.

### 4.5. Blood Sampling

Whole-blood samples for CBC and flow cytometry (FACS) analysis were collected via mandibular vein puncture in Microtainer K2E tubes (365975; Becton Dickinson, Franklin Lakes, NJ, USA) at 7, 14, 21, 28, 35, and 42 d.p.i.

### 4.6. Evaluation of Engraftment

The primary factor affecting donor–graft interactions was assessed by FACS of mouse whole blood samples using antibody conjugates (Table 7). The gating strategy included separation of cell populations based on their forward scatter (FSC) and side scatter (SSC) profiles, isolation of single-cell populations, and analysis of the expression of specific markers in the lymphocyte populations separated by size:

- Determination of the proportion of lymphocytes;
- Determination of the proportion of single lymphocytes;
- Determination of the proportion of viable lymphocytes;
- Determination of the proportion of human lymphocytes (hCD45^+^mCD45^−^) and mouse lymphocytes (hCD45^−^mCD45^+^);
- Determination of the proportion of hT-helper cells in the hT-cell population (Figure S1).

Ch was calculated using the formula:

(1) $$\mathrm{Ch}=\frac{\mathrm{NhCD}45^{+}}{\mathrm{NhCD}45^{+}+\mathrm{NmCD}45^{+}}\times 100\%$$

where

N—number of cells.

Since hT-helper cells are directly responsible for the progression of GVHD [42], their dynamics were monitored throughout the study. The proportion of Th in the total number of hT cells was calculated using the formula:

(2) $$Th=\frac{NhCD3^{+}hCD4^{+}}{NhCD3^{+}hCD4^{+}+NhCD3^{+}hCD4^{-}}\times 100\%$$

where

N—number of cells.

### 4.7. Evaluation of the Effect of IrD and IL-2_ad_ on Mouse Hematopoiesis

WBC, Lymph#, Mon#, Gran#, Lymph%, Mon%, Gran%, RBC, HCT, MCV, MCH, MCHC, RDW, PLT, MPV, PDW, and PCT were determined in whole-blood samples using a Mindray BC-2800Vet automatic hematology analyzer (Mindray Bio-Medical Electronics Co., Ltd., Shenzhen, China).

### 4.8. Pathomorphology

During the necropsy, a macroscopic examination was performed, accompanied by organ weight measurements using an Ohaus Pioneer PX 223 scale (Ohaus, Parsippany, NJ, USA). The following organs were removed for histological preparation: the spleen, lungs, kidneys with adrenal glands, liver, BM with sternum, small and large intestines with mesentery and mesenteric lymph nodes, brain, organs of the reproductive tract, and perithymic tissue. Lungs and kidneys were weighed in pairs. Relative organ weight was calculated using the following formula:

(3) $$Relative\;organ\;weight=\frac{Organ\;weight}{Body\;weight}\times 100\%$$

### 4.9. Histology and Immunohistochemistry

Sections were prepared from formalin-fixed paraffin-embedded tissue and H&E-stained according to standard procedures. Organ and tissue samples were fixed in 10% buffered formalin, dehydrated through a series of isopropyl alcohols of increasing concentration, and embedded in paraffin blocks. Sections of 4–5 μm thickness were prepared from the blocks. Slides were examined using an Optika B-500TPL microscope (Optika Microscopes, Bergamo, Italy) equipped with a photo and video recording system at magnifications of 100× and 400×.

HCs in sections were determined via immunochromogenic detection of 3.3-diaminobenzidine tetrahydrochloride (DAB)-labeled anti-hCD4^+^ antibodies (DF16080; Affinity Bioscience, Zhenjiang, China). Polyperoxidase-anti-Rabbit IgG (E-IR-R215B, Elabscience, Wuhan, China) was used as a secondary antibody. DAB working solution was prepared by diluting DAB Concentrate (E-IR-R215D, Elabscience, Wuhan, China) in DAB Substrate (E-IR-R215E, Elabscience, Wuhan, China) according to the manufacturer’s recommendations.

### 4.10. Statistical Analysis

Statistical data processing was performed using GraphPad Prism 9.0.0 software. Survival analysis was performed using the Kaplan–Meier method, modified for the Mantel–Cox and Gehan–Breslow–Wilcoxon tests. The dynamics of CBC indices and the in vivo interaction of grafts and recipients were subjected to ANOVA, determination of the Spearman correlation coefficient (*rs*), and Tukey’s multiple comparison test. To determine *rs*, data obtained at least 3 control points were used. The interpretation of *rs*-values is presented in Table 8. Data obtained from mice euthanized in the first 14 d.p.i. were not used for statistical processing. The threshold value of the significance level (*p*) was set at 0.05. If the *p*-value was below the threshold value of the significance level, the result was considered statistically significant.

## 5. Conclusions

Using a concentration of 0.8 ± 0.6 × 10^7^ hPBMCs/mouse, we obtained an understanding of the optimal parameters for humanization of NSG and NCG mice, as well as confirmation of the hypothesis that, in the IrD/graft concentration pair, IrD plays a leading role in harming the mice’s welfare. Given the modest progression of GVHD and the high probability of survival, preliminary myeloablation with 1.5 Gy is an acceptable regimen for successful humanization of NCG mice. This is also supported by the maximal Ch (~90%) at the end of the study and the promising increase in Th. Looking at the Ch analysis, mice treated with IL-2ad had better human cell engraftment. However, this still carries the risk of sudden death.

For C-NKG mice, IrD of 1 Gy without IL-2_ad_ is acceptable for long-term studies, as they demonstrated the least progression of GVHD and high survival. They also had moderate Ch and Th (~45% for each) at the end of the study.

High and moderate IrD had a negative effect on the survival of HIS-mice, except for NSG. Although IL-2_ad_ resulted in peak Ch and Th at 21 d.p.i., at the end of the study, HIS-mice untreated with IL-2_ad_ showed higher results. However, IL-2_ad_ significantly reduced survival. Therefore, additional graft stimulation generally had a negative effect on the long-term welfare and survival of HIS-mice.

Detection of hCD4^+^ cells in all target organs and tissues of the HIS-mice also confirms the success of humanization. IL-2_ad_, however, generally had no significant effect on the cytosis of human cells. However, a favorable sign is that the cells were diffusely distributed in the organ parenchyma, without forming large clusters or causing local or extensive foci of inflammation.

Thus, the least impact of IrD and IL-2_ad_ on GVHD progression, survival probability, and the dynamics of key CBC indices was observed in NSG mice. This was also confirmed by the results of the pathological examination, according to which these mice had the fewest incidences of pathological changes. At the end of the study, HIS-mice irradiated with 1 Gy and untreated with IL-2_ad_ NSG had the highest Ch (~65%), and the proportion of circulating Th was ~45%, which is a good indicator of the late progression of GVHD.

On the one hand, the models we developed share all the disadvantages of other hPBMC-mice, the main ones being the relatively early development of the lethal form of GVHD and the inability to reconstitute human lymphoid tissue. On the other hand, bone marrow–liver–thymus-mice (BLT-mice), considered the “gold standard” in HIV research, are very difficult to perform and reproduce [43]. Although mice humanized with human HSCs reconstitute human lymphoid tissue in many organs, the availability of hHSCs is very limited, including for ethical reasons. Moreover, hHSCs are also susceptible to the early development of the lethal GVHD phenotype [44]. At the same time, NSG-1, NSG-2, NSG-5, NCG-3, and C-NKG-1 mice are distinguished by increased resistance to GVHD, a high survival rate, high Ch, and a Th concentration sufficient to model HIV infection. Furthermore, human cells were detected in all target organs of these mice. This allows tracking the distribution of HIV after infection.

Since this study did not use mice of the same strain and of different sexes, it lacked comparative implications regarding potential sex differences. However, the survival analysis results demonstrate that C-NKG mice are particularly susceptible to GVHD. Furthermore, returning to BLT mice, the study by Honeycut et al. noted that the brains of female BLT-NSG mice regenerated a higher number of human B-cells than those of males. Moreover, the number of hT-cells was not dependent on sex or IrD [45].

Determining the reference ranges requires large samples. Even without the need to use numerous animals, individual differences remain, since mice of even the same strain, sex, and age, obtained simultaneously from the same breeder and participating in the study under identical conditions, demonstrate different dynamics of CBC indices, Ch, etc. However, with an individual approach, patterns have been identified that may be useful not only in developing individual antiretroviral therapy protocols but also for modeling HIV infection combined with bacterial diseases, as well as oncological diseases. The possibility of using immune-inflammation indices to predict the clinical outcomes of HIV infection in humans is currently being actively promoted [46]. Of course, unmodified mouse blood cells cannot be infected with HIV. Despite this, the use of immune-inflammation indices in the evaluation of mouse models of HIV infection will facilitate better prediction of the duration of in vivo studies and assessment of their effectiveness. We hope that our study will also encourage other research teams to attach greater importance to immune-inflammation indices in HIS-mice and publish reports on this.

## Figures and Tables

**Table 1:** Results of determining the influence of the type and concentration of the graft on the progression of GVHD.

Mouse Strain	% of Total Variation	*p* Value
NCG	37.46	<0.0001
C-NKG	22.4	0.0034

**Table 2:** Determining the probability of survival.

		Log-Rank (Mantel-Cox) Test	Log-Rank Test for Trend	Gehan–Breslow–Wilcoxon Test
NCG	Chi square	28.68	4.905	24.84
	df	6	1	6
	*p* value	<0.0001	0.0268	0.0004
C-NKG	Chi square	28.44	0.7541	21.09
	df	6	1	6
	*p* value	<0.0001	0.3852	0.0018

**Table 3:** Assessment of the statistical relationship between CBC indices and humanization conditions.

CBC Index	ANOVA			Tukey’s Test		
	NSG	NCG	C-NKG	NSG	NCG	C-NKG
WBC	−	+	−	−	IL-2, 35 d.p.i.	IL-2, 42 d.p.i.
Lymph#	−	+	−	−	−	IL-2, 42 d.p.i.
Mon#	−	+	−	−	IL-2, 42 d.p.i.	IL-2, 35 d.p.i.
Gran#	−	−	−	−	IL-2, 42 d.p.i.	IL-2, 28 d.p.i.
Lymph%	−	−	+	−	IR, 21 d.p.i.	−
Mon%	−	−	+	−	−	−
Gran%	+	−	−	−	IR, 7 d.p.i.	−
RBC	−	−	−	−	−	−
HGB	−	−	−	−	−	−
HCT	−	−	−	−	−	−
MCV	+	+	−	−	IR, 28 d.p.i.	−
MCH	+	+	−	IL-2, 21 d.p.i.	IL-2, 28 d.p.i.	−
MCHC	+	+	−	IR, 7, 28 d.p.i.	IR, 28, 35 d.p.i.; IL-2, 28 d.p.i.	−
RDW	+	+	−		IR, 21, 28, 42; IL-2, 42	
PLT	−	−	−	−	−	−
MPV	−	−	−	−	IR, 28 d.p.i.	IL-2, 42 d.p.i.
PDW	−	−	−	IL-2, 35 d.p.i.	−	−
PCT	−	−	−	IL-2, 35 d.p.i.	IR, 42 d.p.i.	−

+: presence of a statistically significant relationship between IrD, IL-2_ad_, and the dynamics of the index; −: absence of statistically significant relationship between IrD, IL-2_ad_, and dynamics of the index; IL-2: a relationship between the dynamics of the index and IL-2_ad_; IR: a relationship between the dynamics of the index and IrD.

**Table 4:** Correlation between the main characteristics of welfare of HIS-mice.

*rs*-Value	NSG, % of Total				NCG, % of Total						C-NKG, % of Total						
	SURV			EUTH	SURV			EUTH			SURV				EUTH		
	BW	GVHD	Ch	BW	BW	GVHD	Ch	BW	GVHD	Ch	BW	GVHD	Ch	Th	BW	GVHD	Ch
GVHD	92			100	80			100			100				100		
Ch	71	75				100		89	100		80	100			89	100	
Th	71	75									80	100					75
WBC			86								80	100	80		88	75	
Lymph#		75					75	80			80	100	100	100			
Mon#			86			75	75				80	100	80				
Gran#			79			75	75					75	100	100			
RBC									86	100	100	75			75	88	
PLT					75			100	100		100	100			100	100	
MLR						100			100			100					
LMR	83					100					75						
GLR		82										75			83		
PLR											80	100					

Negative correlation is highlighted in red; positive correlation is highlighted in blue.

**Table 5:** The most common macromorphological pathologies.

	Anemia				Atrophy			Hyperplasia of the Spleen
	Spleen	Liver	Kidneys	Myocardium	Reproductive Organs	Spleen	Liver	
NSG	2	1	1	1	-	1	1	3
NSG/IL-2	1	1	1	-	1	1	1	6
NCG	5	4	5	1	7	2	2	9
NCG/IL-2	6	4	6	5	6	1	5	8
C-NKG	8	1	6	5	8	4	8	6
C-NKG/IL-2	11	-	10	3	9	6	11	5

**Table 6:** Characteristics of animal groups.

	IrD, Gy	IL-2_ad_	Mouse Group
Donor 1	1.0	No	NSG-1
		Yes	NSG-2
	1.5	No	NSG-3
		Yes	NSG-4
	2.0	No	NSG-5
		Yes	NSG-6
Donor 2	1.0	No	NCG-1
		Yes	NCG-2
	1.5	No	NCG-3
		Yes	NCG-4
	2.0	No	NCG-5
		Yes	NCG-6
Donor 3	1.0	No	C-NKG-1
		Yes	C-NKG-2
	1.5	No	C-NKG-3
		Yes	C-NKG-4
	2.0	No	C-NKG-5
		Yes	C-NKG-6
None		None	Control

**Table 7:** Antibody conjugates for flow cytometry.

Dye/Antibody Conjugate	Catalogue Number	Manufacturer
7-AAD Staining Solution	130-111-568	Miltenyi Biotec, Bergisch Gladbach, Germany
FITC Anti-Human CD45 Antibody [HI30]	E-AB-F1137C	Elabscience Biotechnology Co., Ltd., Wuhan, China
EV450 Anti-Mouse CD45 Antibody [30-F11]	E-AB-F1136Q	Elabscience Biotechnology Co., Ltd., Wuhan, China
PE/Cyanine7 Anti-Human CD4 Antibody [RPA-T4]	E-AB-F1109H	Elabscience Biotechnology Co., Ltd., Wuhan, China
PE/TR Anti-Human CD3 Antibody [OKT-3]	E-AB-F1001P	Elabscience Biotechnology Co., Ltd., Wuhan, China

**Table 8:** Interpretation of *rs*-values.

*rs-*Value	Interpretation of Correlation
0.75 to 1.00 (−0.75 to −1.00)	Very high positive (negative)
0.50 to 0.75 (−0.50 to −0.75)	High positive (negative)
0.25 to 0.50 (−0.25 to− 0.50)	Moderate positive (negative)
0.00 to 0.25 (0.00 to −0.25)	Low positive (negative)

## Data Availability

The authors confirm that the data supporting the findings of this study are available within the manuscript.

## Supplementary Materials

The following supporting information can be downloaded at: https://www.mdpi.com/article/10.3390/ijms27146266/s1.

## Abbreviations

The following abbreviations are used in this manuscript:

HIV	Human immunodeficiency virus.
CBC	Complete blood count.
BW	Body weight.
GVHD	Graft-versus-host disease.
HIS	Human immune system.
IL-2_ad_	Administration of interleukin-2.
MHC	Major histocompatibility complex.
HLA	Human leukocyte antigen.
hPBMCs-mice	Engrafted with human peripheral blood mononuclear cells HIS-mice.
d.p.i.	Days post-injection of human cells.
HSC	Hematopoietic stem cells.
MLR	Monocyte-to-lymphocyte ratio.
LMR	Lymphocyte-to-monocyte ratio.
GLR	Granulocyte-to-lymphocyte ratio.
PLR	Platelet-to-lymphocyte ratio.
Ch	Degree of chimerism.
ANOVA	Two-way analysis of variance.
IrD	Irradiation dose.
Th	hT-helper cell.
WBC	Absolute white blood cell count.
Lymph#	Absolute lymphocyte count.
Mon#	Absolute monocyte count.
Gran#	Absolute granulocyte count.
Lymph%	Relative lymphocyte count.
Mon%	Relative monocyte count.
Gran%	Relative granulocyte count.
RBC	Absolute red blood cell count.
HGB	Hemoglobin count.
HCT	Hematocrit.
MCV	Mean corpuscular volume.
MCH	Mean corpuscular hemoglobin.
MCHC	Mean corpuscular hemoglobin concentration.
RDW	Red cell distribution width.
PLT	Absolute platelet count.
MPV	Mean platelet volume.
PDW	Platelet distribution width.
PCT	Plateletcrit.
BM	Bone marrow.
H&E	Hematoxylin and eosin.
DAB	3.3-diaminobenzidine tetrahydrochloride.
BLT	Bone marrow–liver–thymus.
