# Supplementary material for: Interleukin-2-Mediated Engraftment of Human Peripheral Blood Mononuclear Cells in Immunodeficient Mice to Develop a Model of HIV Infection: New Criteria for Engraftment Monitoring
Source: Int J Mol Sci. 2026 Jul 14;27(14):6266. doi: 10.3390/ijms27146266 (PMC13409855; doi:10.3390/ijms27146266)
Supplement: Supplementary file 1 [file ijms-27-06266-s001.zip › Supplementary files/Figure S1.pdf]

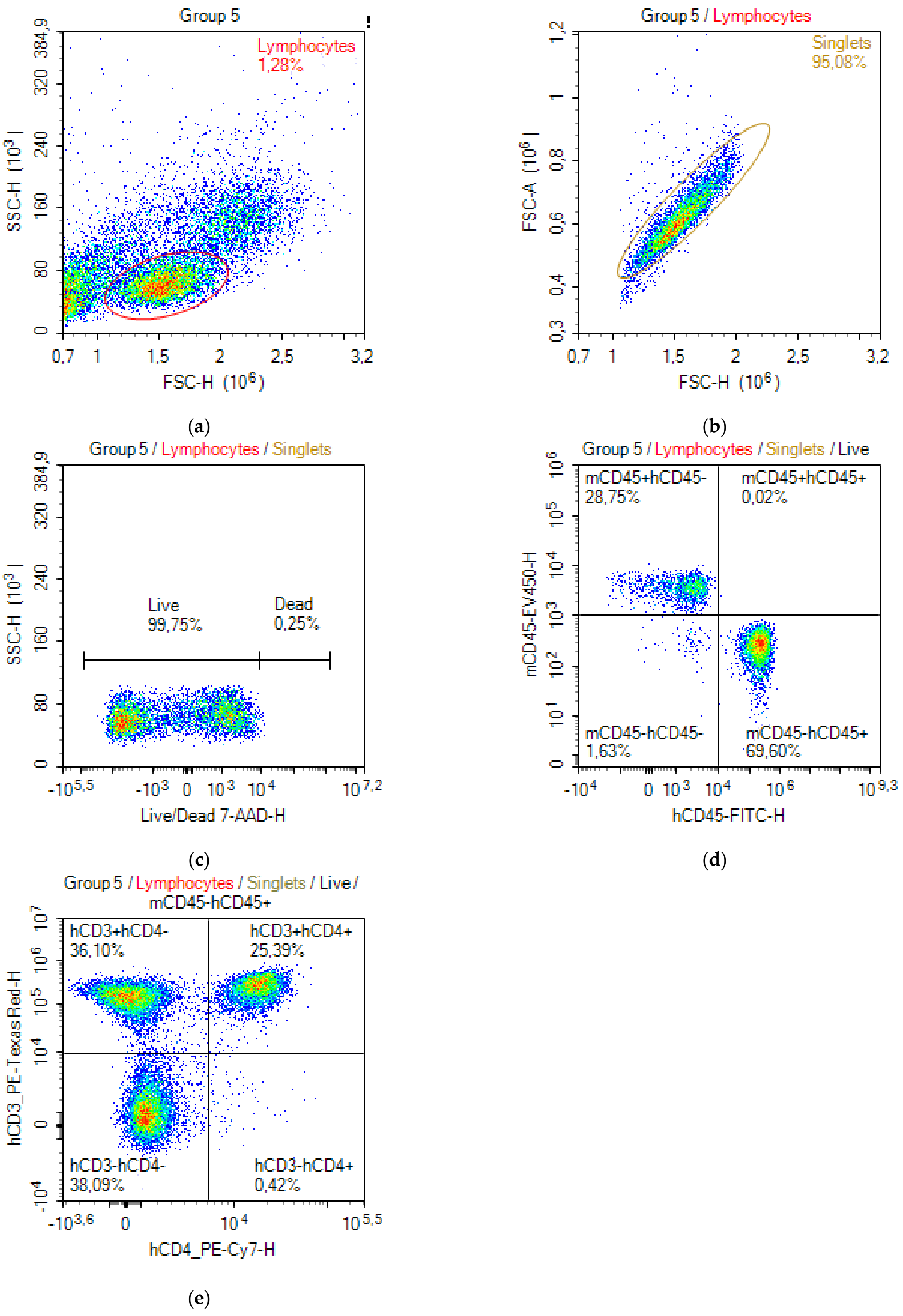

Figure S1. Strategy for defining hT-cell and hT-helper cell populations relative to mouse lymphocytes. The fluorescence data are displayed on a biexponential scale to accurately represent the compensated 7-AAD negative population. (**a**) Determination of the lymphocyte fraction by FSC and SSC; (**b**) Single-cell cytometry; (**c**) Determination of viable cells among singlets; (**d**) Separation of populations of hT-cells ($mCD45^{-}hCD45^{+}$) and mouse lymphocytes ($mCD45^{+}hCD45^{-}$); (**e**) Determination of the fraction of hT-helper cells ($hCD3^{+}hCD4^{+}$) in the hT-cells population.
