# Supplementary material for: Interleukin-2-Mediated Engraftment of Human Peripheral Blood Mononuclear Cells in Immunodeficient Mice to Develop a Model of HIV Infection: New Criteria for Engraftment Monitoring
Source: Int J Mol Sci. 2026 Jul 14;27(14):6266. doi: 10.3390/ijms27146266 (PMC13409855; doi:10.3390/ijms27146266)
Supplement: Supplementary file 1 [file ijms-27-06266-s001.zip › Supplementary files/Figure S4.pdf]

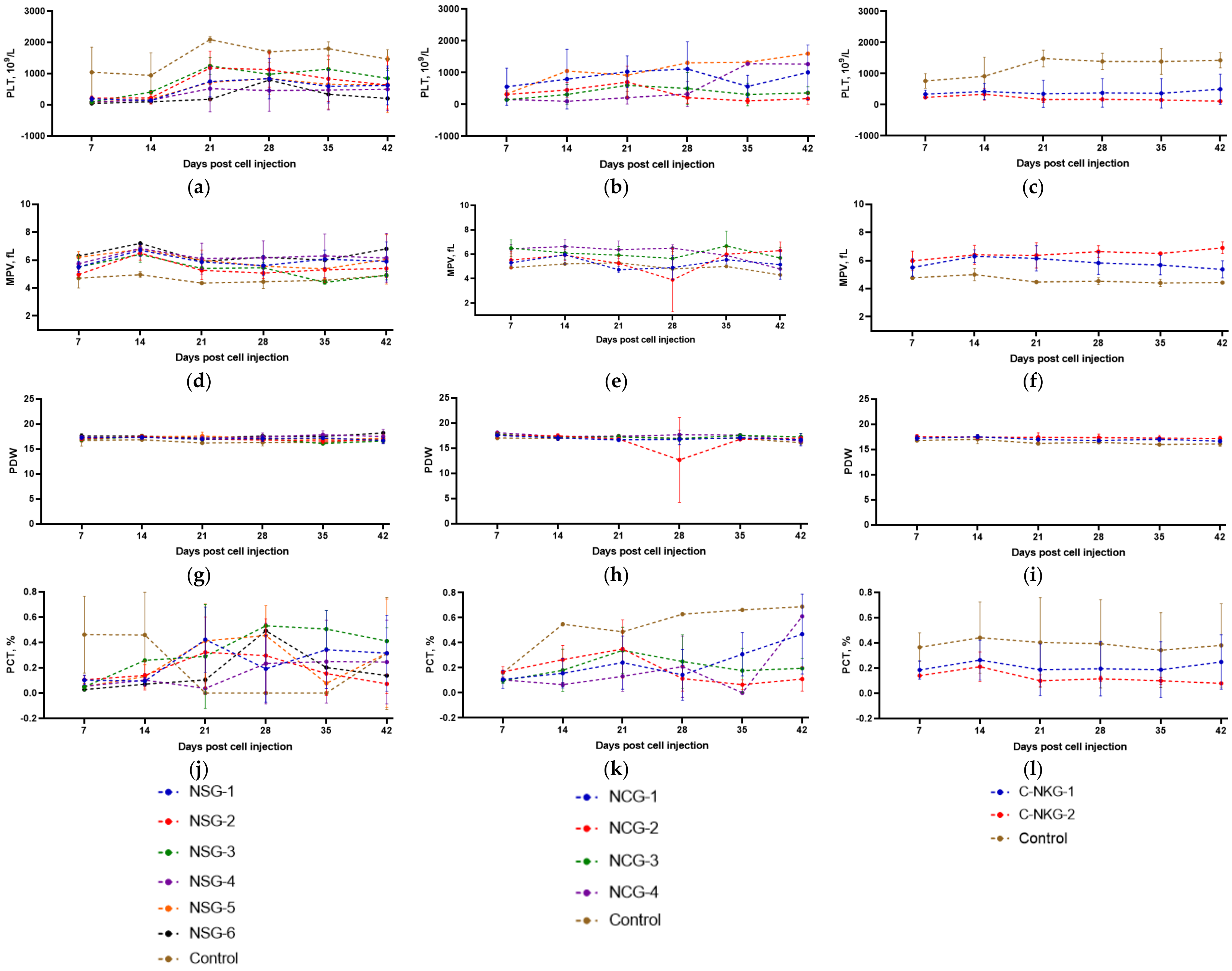

Figure S4. Effect of the IrD and IL-2_{ad} on the dynamics of platelet indices in: (**a**, **d**, **g**, **j**) NSG mice; (**b**, **e**, **h**, **k**) NCG mice; (**c**, **f**, **i**, **l**) C-NKG mice.
