# Supplementary material for: Interleukin-2-Mediated Engraftment of Human Peripheral Blood Mononuclear Cells in Immunodeficient Mice to Develop a Model of HIV Infection: New Criteria for Engraftment Monitoring
Source: Int J Mol Sci. 2026 Jul 14;27(14):6266. doi: 10.3390/ijms27146266 (PMC13409855; doi:10.3390/ijms27146266)
Supplement: Supplementary file 1 [file ijms-27-06266-s001.zip › Supplementary files/Figure S5.pdf]

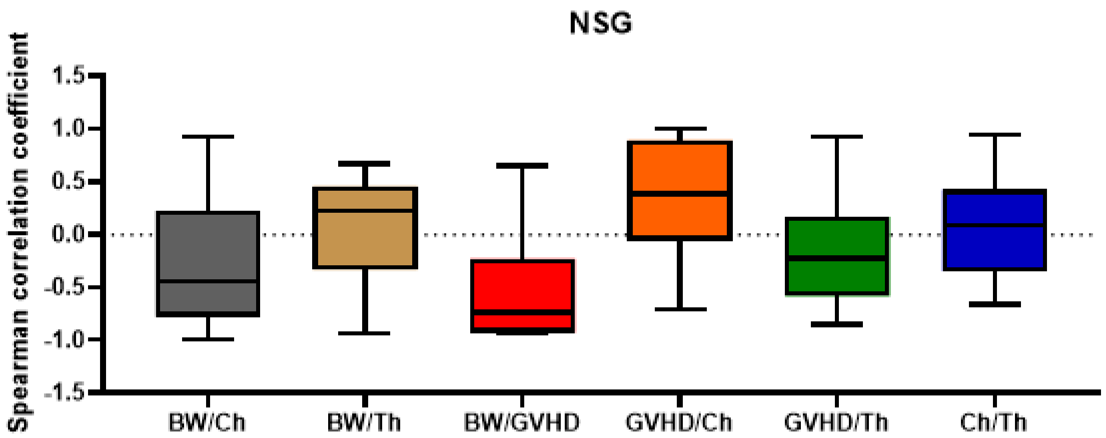

(**a**)

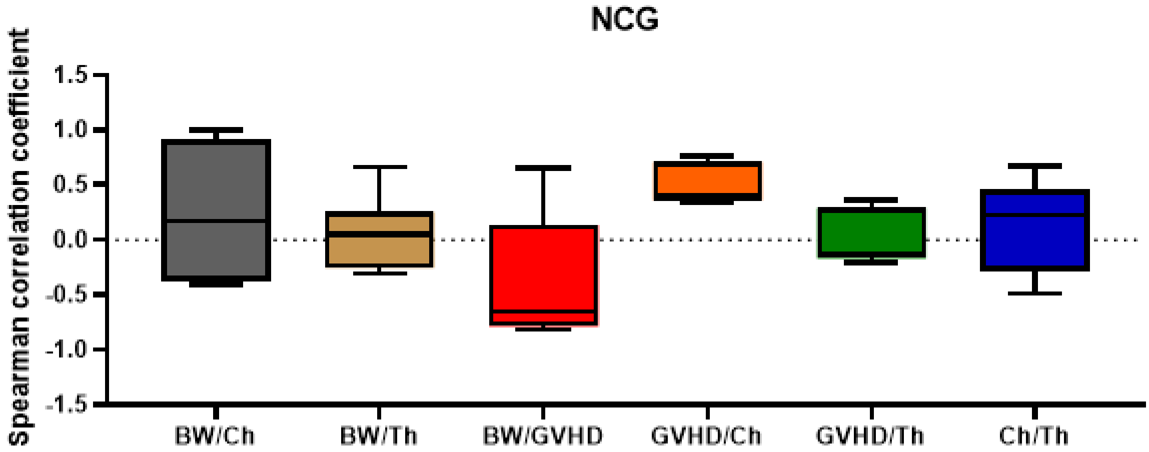

(**b**)

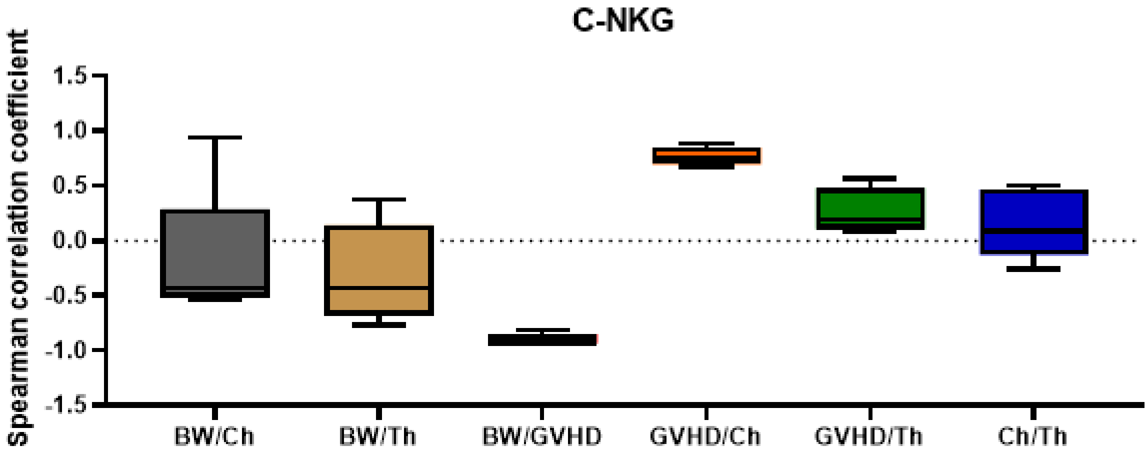

(**c**)

Figure S5. Correlation between BW, GVHD, Ch, and Th in: (**a**) NSG_{SURV} mice; (**b**) NCG_{SURV} mice; (**c**) $C\text{-}NKG_{SURV}$ mice.
