# Supplementary material for: Interleukin-2-Mediated Engraftment of Human Peripheral Blood Mononuclear Cells in Immunodeficient Mice to Develop a Model of HIV Infection: New Criteria for Engraftment Monitoring
Source: Int J Mol Sci. 2026 Jul 14;27(14):6266. doi: 10.3390/ijms27146266 (PMC13409855; doi:10.3390/ijms27146266)
Supplement: Supplementary file 1 [file ijms-27-06266-s001.zip › Supplementary files/Table S10.pdf]

Table S10. Number of mice included in the analysis at each control point.

Check point, d.p.i.	NSG mice group							NCG mice group							C-NKG mice group						
	**1**	**2**	**3**	**4**	**5**	**6**	**Control**	**1**	**2**	**3**	**4**	**5**	**6**	**Control**	**1**	**2**	**3**	**4**	**5**	**6**	**Control**
0	3	3	3	3	3	3	3	4	4	4	4	4	4	4	4	4	4	4	4	4	4
7	3	3	3	3	3	3	3	4	4	4	4	3	4	1[1]	4	4	4	4	4	4	4
14	3	3	2	3	3	1	3	4	4	4	4	3	3	1	4	4	4	4	4	4	4
21	3	3	2	3	3	1	3	3	4	4	4	2	1	1	4	3	3	2	0	3	4
28	3	3	2	3	3	1	3	2	4	4	2	0	1	1	4	3	2	2	0	0	4
35	3	3	2	2	3	1	3	2	3	4	1	0	1	1	4	3	0	0	0	0	4
42	3	3	2	2	3	1	3	2	2	3	1	0	1	1	3	2	0	0	0	0	4

[1] Mice were excluded for reasons unrelated to the study (mutual injuries resulting from unmotivated aggression), but this did not affect the analysis results and conclusions.
