# Supplementary material for: Interleukin-2-Mediated Engraftment of Human Peripheral Blood Mononuclear Cells in Immunodeficient Mice to Develop a Model of HIV Infection: New Criteria for Engraftment Monitoring
Source: Int J Mol Sci. 2026 Jul 14;27(14):6266. doi: 10.3390/ijms27146266 (PMC13409855; doi:10.3390/ijms27146266)
Supplement: Supplementary file 1 [file ijms-27-06266-s001.zip › Supplementary files/Table S8.pdf]

Table S8. Micromorphology of the brain

Group number	NSG mice		NCG mice		C-NKG mice	
	**H&E staining, x400**	**DAB detection, x400**	**H&E staining, x400**	**DAB detection, x400**	**H&E staining, x400**	**DAB detection, x400**
1	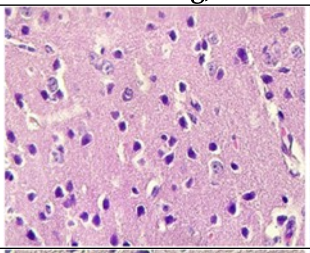	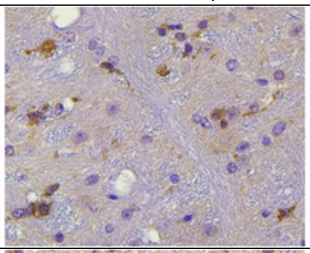	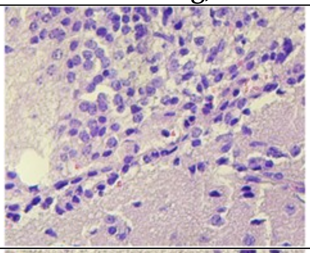	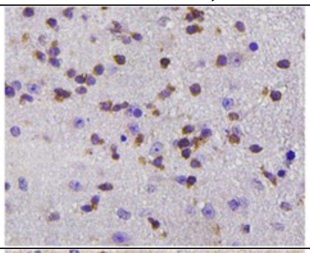	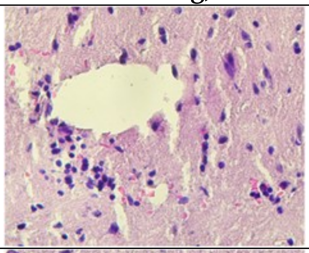	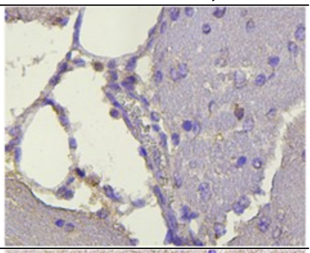
2	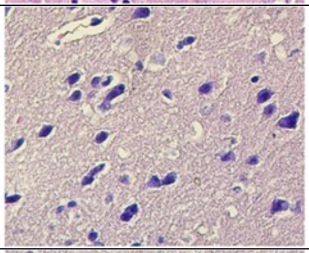	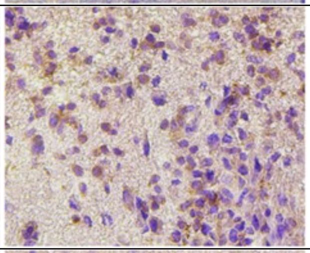	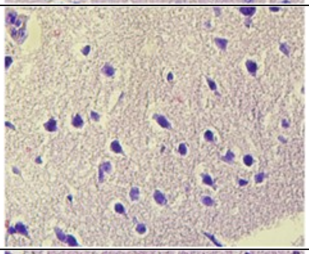	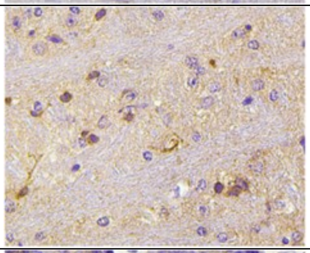	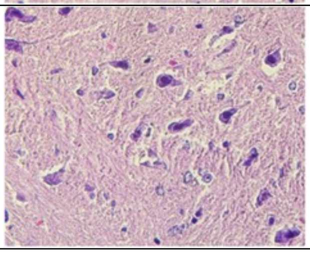	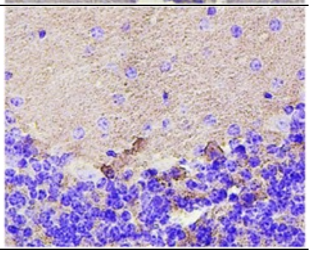
3	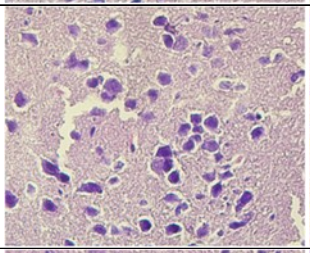	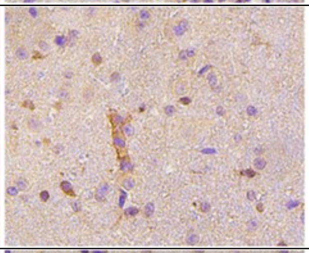	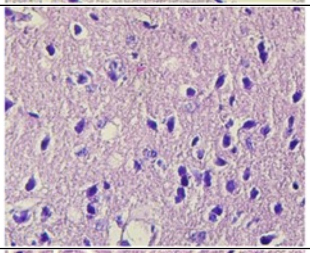	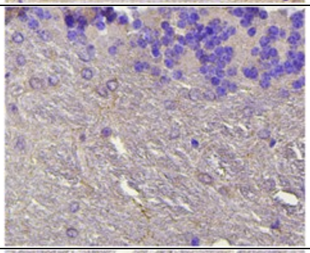		
4	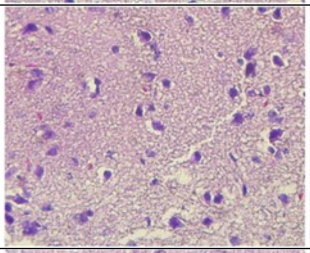	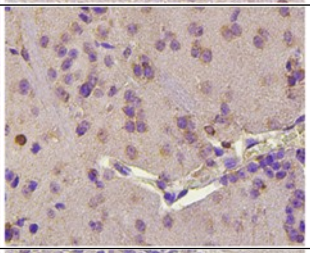	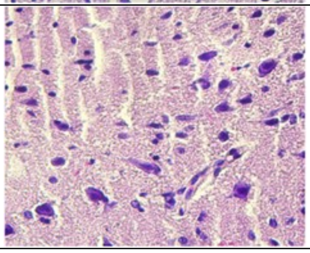	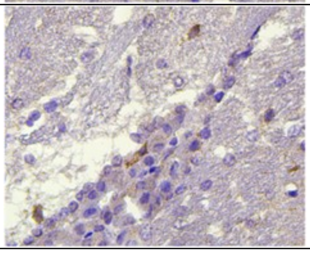		
5	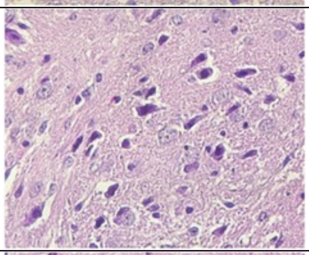	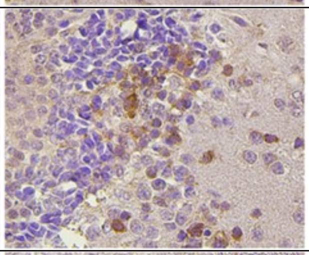				
6	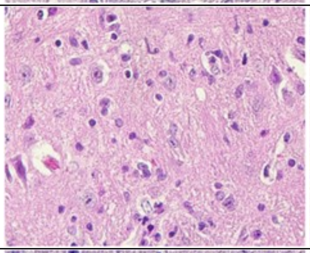	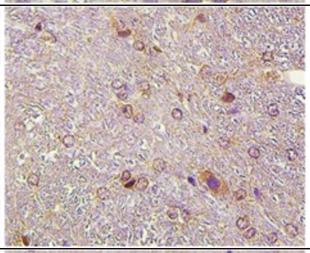				
Control	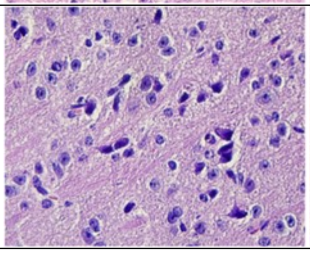	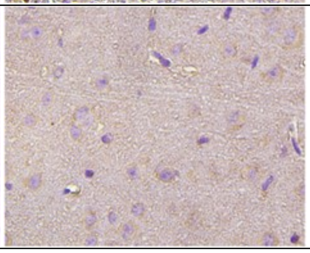	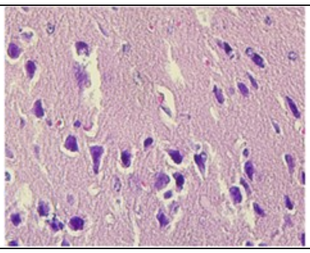	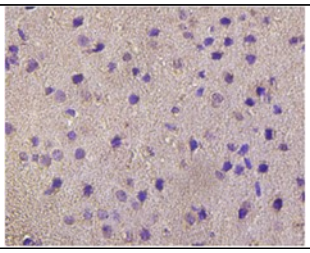	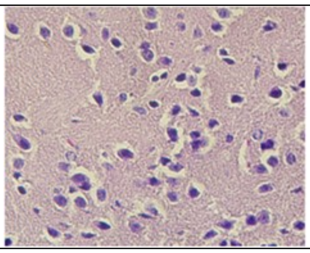	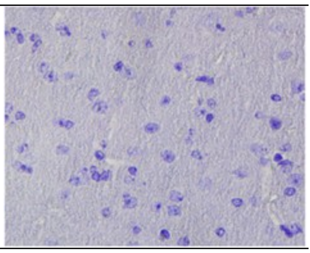
