# Supplementary figures and images for: Interleukin-2-Mediated Engraftment of Human Peripheral Blood Mononuclear Cells in Immunodeficient Mice to Develop a Model of HIV Infection: New Criteria for Engraftment Monitoring
Source: Int J Mol Sci. 2026 Jul 14;27(14):6266. doi: 10.3390/ijms27146266 (PMC13409855; doi:10.3390/ijms27146266)

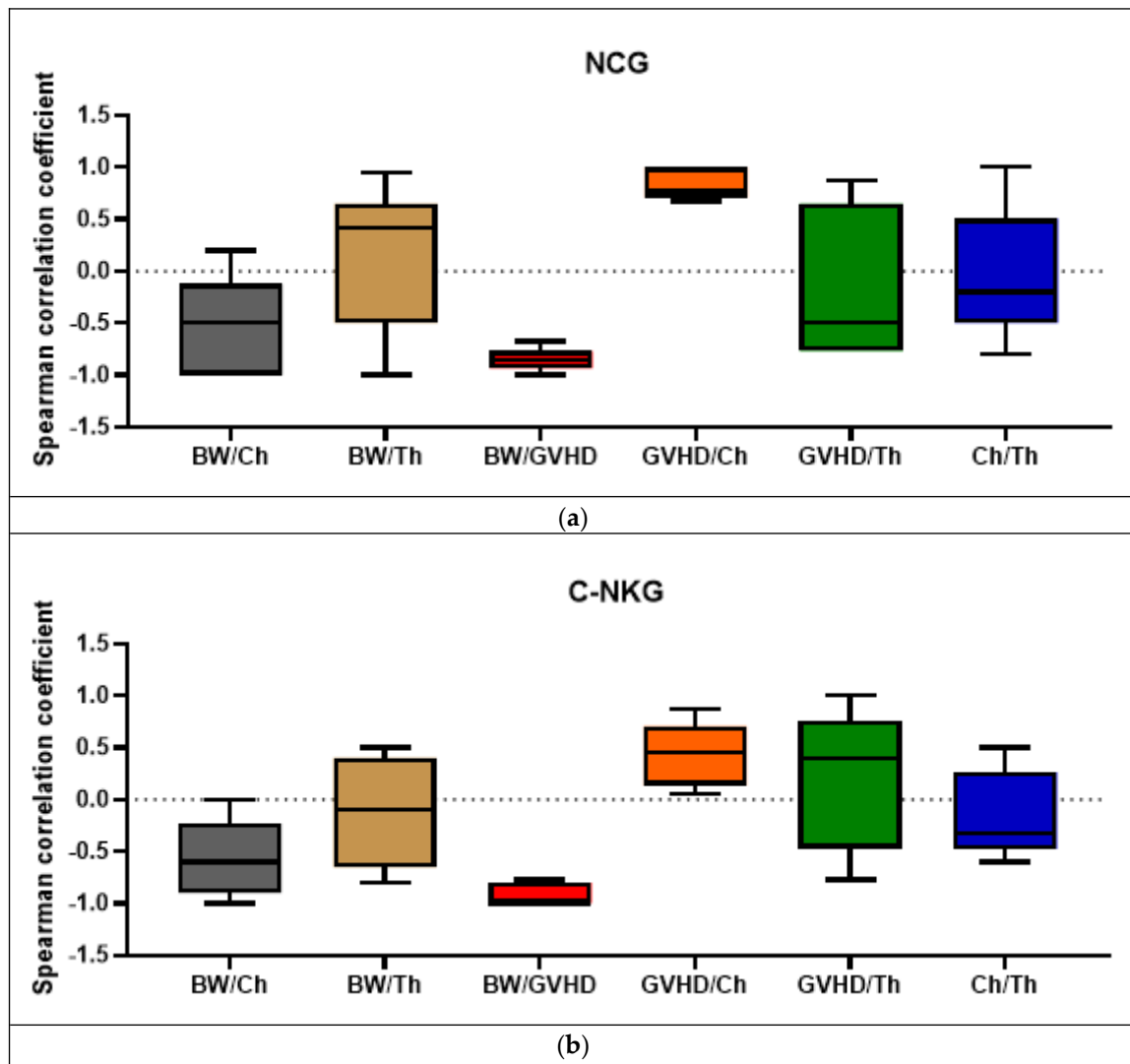

**Figure S6.** Correlation between GVHD, Ch, and Th in: **(a)** NCG<sub>EUTH</sub> mice; **(b)** C-NKG<sub>EUTH</sub> mice.

Supplement: Supplementary file 1 [file ijms-27-06266-s001.zip › Supplementary files/Figure S6.pdf]
